# Supplementary material for: An evaluation of trace elements and oxidative stress in patients with benign paroxysmal positional vertigo
Source: PLoS One. 2023 Feb 22;18(2):e0277960. doi: 10.1371/journal.pone.0277960 (PMC9946263; doi:10.1371/journal.pone.0277960)
Supplement: S2 File — (DOCX) [file pone.0277960.s002.docx]

**Project Name:** alanya huseyin gunizi

**Dataset(s):** VertigoSPSSData.sav

**User(s):** AHMET ÖZTÜRK

**Module:** Independent Two Samples t - **Panel:** Parametric Tests

‘Independent Two Samples t Test’ module estimates the means of two independent populations. Using this module, you can identify whether the mean of the differences between two independent samples differs from 0 (or a test value). You can display the results with interactive error bars and boxplots. You can also check whether the data normality assumption is met and the variances are homogeneous to each other.

**Dataset:** VertigoSPSSData.sav

**Selected Variable(s):**

**Response variable(s):** Age, Zn, Cu, TotalThiol, NativeThiol, Disulfide, ReducedThiol, OxidizedThiol, ThiolOxRedRatio

**Group variable(s):** Group

**Test proportion:** 0

**Show**

**Response variable:** Age

**Group variable:** Group

**Descriptive Statistics**

| **Group** | **N** | **Mean** | **Std.Dev.** | **S.E.Mean** | **Mean (Lower)** | **Mean (Upper)** |
| --- | --- | --- | --- | --- | --- | --- |
| Control (A) | 66 | 44.7727 | 13.243 | 1.6301 | 41.5172 | 48.0283 |
| vertigo (B) | 66 | 45.7576 | 15.1455 | 1.8643 | 42.0343 | 49.4808 |
| Diff. (A - B)(t-Test) |  | -0.9848 |  | 2.4764 | -5.8842 | 3.9145 |
| Diff. (A - B)(Welch) |  | -0.9848 |  | 2.4764 | -5.885 | 3.9153 |

**Test Results & Statistics**

**Tests**

Student's t-test

| **Variable** | **d.f.** | **Test statistic (t)** | **p value** | **Test variance** | **Estimate** |
| --- | --- | --- | --- | --- | --- |
| Diff. (A - B) | 130 | -0.3977 | 0.692 | 0 | -0.9848 |

There is no statistically significant difference between the **Group** categories in terms of **Age** mean values **(p=0.692)**.

Welch

| **Variable** | **d.f.** | **Test statistic (t)** | **p value** | **Test variance** | **Estimate** |
| --- | --- | --- | --- | --- | --- |
| Diff. (A - B) | 127.726 | -0.3977 | 0.692 | 0 | -0.9848 |

There is no statistically significant difference between the **Group** categories in terms of **Age** mean values **(p=0.692)**.

**Show**

**Response variable:** Zn

**Group variable:** Group

**Descriptive Statistics**

| **Group** | **N** | **Mean** | **Std.Dev.** | **S.E.Mean** | **Mean (Lower)** | **Mean (Upper)** |
| --- | --- | --- | --- | --- | --- | --- |
| Control (A) | 66 | 85.8606 | 7.6114 | 0.9369 | 83.9895 | 87.7317 |
| vertigo (B) | 66 | 87.147 | 13.2657 | 1.6329 | 83.8858 | 90.4081 |
| Diff. (A - B)(t-Test) |  | -1.2864 |  | 1.8826 | -5.0108 | 2.4381 |
| Diff. (A - B)(Welch) |  | -1.2864 |  | 1.8826 | -5.0198 | 2.4471 |

**Test Results & Statistics**

**Tests**

Student's t-test

| **Variable** | **d.f.** | **Test statistic (t)** | **p value** | **Test variance** | **Estimate** |
| --- | --- | --- | --- | --- | --- |
| Diff. (A - B) | 130 | -0.6833 | 0.496 | 0 | -1.2864 |

There is no statistically significant difference between the **Group** categories in terms of **Zn** mean values **(p=0.496)**.

Welch

| **Variable** | **d.f.** | **Test statistic (t)** | **p value** | **Test variance** | **Estimate** |
| --- | --- | --- | --- | --- | --- |
| Diff. (A - B) | 103.6124 | -0.6833 | 0.496 | 0 | -1.2864 |

There is no statistically significant difference between the **Group** categories in terms of **Zn** mean values **(p=0.496)**.

**Show**

**Response variable:** Cu

**Group variable:** Group

**Descriptive Statistics**

| **Group** | **N** | **Mean** | **Std.Dev.** | **S.E.Mean** | **Mean (Lower)** | **Mean (Upper)** |
| --- | --- | --- | --- | --- | --- | --- |
| Control (A) | 66 | 135.7727 | 42.2603 | 5.2019 | 125.3839 | 146.1616 |
| vertigo (B) | 66 | 119.6061 | 47.632 | 5.8631 | 107.8967 | 131.3155 |
| Diff. (A - B)(t-Test) |  | 16.1667 |  | 7.8381 | 0.66 | 31.6734 |
| Diff. (A - B)(Welch) |  | 16.1667 |  | 7.8381 | 0.6579 | 31.6754 |

**Test Results & Statistics**

**Tests**

Student's t-test

| **Variable** | **d.f.** | **Test statistic (t)** | **p value** | **Test variance** | **Estimate** |
| --- | --- | --- | --- | --- | --- |
| Diff. (A - B) | 130 | 2.0626 | 0.041 | 0 | 16.1667 |

There is a statistically significant difference between the **Group** categories in terms of **Cu** mean values. Mean **Cu** value in the **Control (A) (135.7727)** category is found to be higher than the **vertigo (B) (119.6061)** category **(p=0.041)**.

Welch

| **Variable** | **d.f.** | **Test statistic (t)** | **p value** | **Test variance** | **Estimate** |
| --- | --- | --- | --- | --- | --- |
| Diff. (A - B) | 128.1821 | 2.0626 | 0.041 | 0 | 16.1667 |

There is a statistically significant difference between the **Group** categories in terms of **Cu** mean values. Mean **Cu** value in the **Control (A) (135.7727)** category is found to be higher than the **vertigo (B) (119.6061)** category **(p=0.041)**.

**Show**

**Response variable:** TotalThiol

**Group variable:** Group

**Descriptive Statistics**

| **Group** | **N** | **Mean** | **Std.Dev.** | **S.E.Mean** | **Mean (Lower)** | **Mean (Upper)** |
| --- | --- | --- | --- | --- | --- | --- |
| Control (A) | 66 | 611.7333 | 84.8465 | 10.4439 | 590.8755 | 632.5912 |
| vertigo (B) | 66 | 535.0273 | 154.7183 | 19.0445 | 496.9928 | 573.0618 |
| Diff. (A - B)(t-Test) |  | 76.7061 |  | 21.7202 | 33.7352 | 119.6769 |
| Diff. (A - B)(Welch) |  | 76.7061 |  | 21.7202 | 33.6182 | 119.7939 |

**Test Results & Statistics**

**Tests**

Student's t-test

| **Variable** | **d.f.** | **Test statistic (t)** | **p value** | **Test variance** | **Estimate** |
| --- | --- | --- | --- | --- | --- |
| Diff. (A - B) | 130 | 3.5316 | <0.001 | 0 | 76.7061 |

There is a statistically significant difference between the **Group** categories in terms of **TotalThiol** mean values. Mean **TotalThiol** value in the **Control (A) (611.7333)** category is found to be higher than the **vertigo (B) (535.0273)** category **(p<0.001)**.

Welch

| **Variable** | **d.f.** | **Test statistic (t)** | **p value** | **Test variance** | **Estimate** |
| --- | --- | --- | --- | --- | --- |
| Diff. (A - B) | 100.853 | 3.5316 | <0.001 | 0 | 76.7061 |

There is a statistically significant difference between the **Group** categories in terms of **TotalThiol** mean values. Mean **TotalThiol** value in the **Control (A) (611.7333)** category is found to be higher than the **vertigo (B) (535.0273)** category **(p<0.001)**.

**Show**

**Response variable:** NativeThiol

**Group variable:** Group

**Descriptive Statistics**

| **Group** | **N** | **Mean** | **Std.Dev.** | **S.E.Mean** | **Mean (Lower)** | **Mean (Upper)** |
| --- | --- | --- | --- | --- | --- | --- |
| Control (A) | 66 | 551.2788 | 89.3462 | 10.9978 | 529.3147 | 573.2428 |
| vertigo (B) | 66 | 328.497 | 98.1599 | 12.0826 | 304.3663 | 352.6277 |
| Diff. (A - B)(t-Test) |  | 222.7818 |  | 16.3383 | 190.4584 | 255.1052 |
| Diff. (A - B)(Welch) |  | 222.7818 |  | 16.3383 | 190.4557 | 255.1079 |

**Test Results & Statistics**

**Tests**

Student's t-test

| **Variable** | **d.f.** | **Test statistic (t)** | **p value** | **Test variance** | **Estimate** |
| --- | --- | --- | --- | --- | --- |
| Diff. (A - B) | 130 | 13.6355 | <0.001 | 0 | 222.7818 |

There is a statistically significant difference between the **Group** categories in terms of **NativeThiol** mean values. Mean **NativeThiol** value in the **Control (A) (551.2788)** category is found to be higher than the **vertigo (B) (328.497)** category **(p<0.001)**.

Welch

| **Variable** | **d.f.** | **Test statistic (t)** | **p value** | **Test variance** | **Estimate** |
| --- | --- | --- | --- | --- | --- |
| Diff. (A - B) | 128.8661 | 13.6355 | <0.001 | 0 | 222.7818 |

There is a statistically significant difference between the **Group** categories in terms of **NativeThiol** mean values. Mean **NativeThiol** value in the **Control (A) (551.2788)** category is found to be higher than the **vertigo (B) (328.497)** category **(p<0.001)**.

**Show**

**Response variable:** Disulfide

**Group variable:** Group

**Descriptive Statistics**

| **Group** | **N** | **Mean** | **Std.Dev.** | **S.E.Mean** | **Mean (Lower)** | **Mean (Upper)** |
| --- | --- | --- | --- | --- | --- | --- |
| Control (A) | 66 | 30.25 | 13.7514 | 1.6927 | 26.8695 | 33.6305 |
| vertigo (B) | 66 | 104.2227 | 41.224 | 5.0743 | 94.0886 | 114.3568 |
| Diff. (A - B)(t-Test) |  | -73.9727 |  | 5.3492 | -84.5555 | -63.39 |
| Diff. (A - B)(Welch) |  | -73.9727 |  | 5.3492 | -84.6194 | -63.326 |

**Test Results & Statistics**

**Tests**

Student's t-test

| **Variable** | **d.f.** | **Test statistic (t)** | **p value** | **Test variance** | **Estimate** |
| --- | --- | --- | --- | --- | --- |
| Diff. (A - B) | 130 | -13.8288 | <0.001 | 0 | -73.9727 |

There is a statistically significant difference between the **Group** categories in terms of **Disulfide** mean values. Mean **Disulfide** value in the **vertigo (B) (104.2227)** category is found to be higher than the **Control (A) (30.25)** category **(p<0.001)**.

Welch

| **Variable** | **d.f.** | **Test statistic (t)** | **p value** | **Test variance** | **Estimate** |
| --- | --- | --- | --- | --- | --- |
| Diff. (A - B) | 79.2887 | -13.8288 | <0.001 | 0 | -73.9727 |

There is a statistically significant difference between the **Group** categories in terms of **Disulfide** mean values. Mean **Disulfide** value in the **vertigo (B) (104.2227)** category is found to be higher than the **Control (A) (30.25)** category **(p<0.001)**.

**Show**

**Response variable:** ReducedThiol

**Group variable:** Group

**Descriptive Statistics**

| **Group** | **N** | **Mean** | **Std.Dev.** | **S.E.Mean** | **Mean (Lower)** | **Mean (Upper)** |
| --- | --- | --- | --- | --- | --- | --- |
| Control (A) | 66 | 89.9076 | 4.9571 | 0.6102 | 88.689 | 91.1262 |
| vertigo (B) | 66 | 61.747 | 9.5312 | 1.1732 | 59.4039 | 64.09 |
| Diff. (A - B)(t-Test) |  | 28.1606 |  | 1.3224 | 25.5444 | 30.7768 |
| Diff. (A - B)(Welch) |  | 28.1606 |  | 1.3224 | 25.5363 | 30.7849 |

**Test Results & Statistics**

**Tests**

Student's t-test

| **Variable** | **d.f.** | **Test statistic (t)** | **p value** | **Test variance** | **Estimate** |
| --- | --- | --- | --- | --- | --- |
| Diff. (A - B) | 130 | 21.2951 | <0.001 | 0 | 28.1606 |

There is a statistically significant difference between the **Group** categories in terms of **ReducedThiol** mean values. Mean **ReducedThiol** value in the **Control (A) (89.9076)** category is found to be higher than the **vertigo (B) (61.747)** category **(p<0.001)**.

Welch

| **Variable** | **d.f.** | **Test statistic (t)** | **p value** | **Test variance** | **Estimate** |
| --- | --- | --- | --- | --- | --- |
| Diff. (A - B) | 97.7678 | 21.2951 | <0.001 | 0 | 28.1606 |

There is a statistically significant difference between the **Group** categories in terms of **ReducedThiol** mean values. Mean **ReducedThiol** value in the **Control (A) (89.9076)** category is found to be higher than the **vertigo (B) (61.747)** category **(p<0.001)**.

**Show**

**Response variable:** OxidizedThiol

**Group variable:** Group

**Descriptive Statistics**

| **Group** | **N** | **Mean** | **Std.Dev.** | **S.E.Mean** | **Mean (Lower)** | **Mean (Upper)** |
| --- | --- | --- | --- | --- | --- | --- |
| Control (A) | 66 | 5.0561 | 2.4823 | 0.3055 | 4.4458 | 5.6663 |
| vertigo (B) | 66 | 19.3333 | 4.1894 | 0.5157 | 18.3034 | 20.3632 |
| Diff. (A - B)(t-Test) |  | -14.2773 |  | 0.5994 | -15.4631 | -13.0914 |
| Diff. (A - B)(Welch) |  | -14.2773 |  | 0.5994 | -15.4657 | -13.0888 |

**Test Results & Statistics**

**Tests**

Student's t-test

| **Variable** | **d.f.** | **Test statistic (t)** | **p value** | **Test variance** | **Estimate** |
| --- | --- | --- | --- | --- | --- |
| Diff. (A - B) | 130 | -23.8191 | <0.001 | 0 | -14.2773 |

There is a statistically significant difference between the **Group** categories in terms of **OxidizedThiol** mean values. Mean **OxidizedThiol** value in the **vertigo (B) (19.3333)** category is found to be higher than the **Control (A) (5.0561)** category **(p<0.001)**.

Welch

| **Variable** | **d.f.** | **Test statistic (t)** | **p value** | **Test variance** | **Estimate** |
| --- | --- | --- | --- | --- | --- |
| Diff. (A - B) | 105.6307 | -23.8191 | <0.001 | 0 | -14.2773 |

There is a statistically significant difference between the **Group** categories in terms of **OxidizedThiol** mean values. Mean **OxidizedThiol** value in the **vertigo (B) (19.3333)** category is found to be higher than the **Control (A) (5.0561)** category **(p<0.001)**.

**Show**

**Response variable:** ThiolOxRedRatio

**Group variable:** Group

**Descriptive Statistics**

| **Group** | **N** | **Mean** | **Std.Dev.** | **S.E.Mean** | **Mean (Lower)** | **Mean (Upper)** |
| --- | --- | --- | --- | --- | --- | --- |
| Control (A) | 66 | 2243.6712 | 1067.3318 | 131.3795 | 1981.2883 | 2506.0541 |
| vertigo (B) | 66 | 343.8712 | 125.3679 | 15.4317 | 313.0519 | 374.6905 |
| Diff. (A - B)(t-Test) |  | 1899.8 |  | 132.2827 | 1638.0946 | 2161.5054 |
| Diff. (A - B)(Welch) |  | 1899.8 |  | 132.2827 | 1635.7477 | 2163.8523 |

**Test Results & Statistics**

**Tests**

Student's t-test

| **Variable** | **d.f.** | **Test statistic (t)** | **p value** | **Test variance** | **Estimate** |
| --- | --- | --- | --- | --- | --- |
| Diff. (A - B) | 130 | 14.3617 | <0.001 | 0 | 1899.8 |

There is a statistically significant difference between the **Group** categories in terms of **ThiolOxRedRatio** mean values. Mean **ThiolOxRedRatio** value in the **Control (A) (2243.6712)** category is found to be higher than the **vertigo (B) (343.8712)** category **(p<0.001)**.

Welch

| **Variable** | **d.f.** | **Test statistic (t)** | **p value** | **Test variance** | **Estimate** |
| --- | --- | --- | --- | --- | --- |
| Diff. (A - B) | 66.7932 | 14.3617 | <0.001 | 0 | 1899.8 |

There is a statistically significant difference between the **Group** categories in terms of **ThiolOxRedRatio** mean values. Mean **ThiolOxRedRatio** value in the **Control (A) (2243.6712)** category is found to be higher than the **vertigo (B) (343.8712)** category **(p<0.001)**.

**Module:** ROC Analysis - **Panel:** Medical Statistics

In this module, you can perform a ROC analysis to predict the performance of a diagnostic test. You can calculate ROC statistics and ROC coordinates and partial AUC. Moreover, you can perform multiple comparison test and predict optimal cutoff points for diagnostic tests. You can also create an interactive ROC curve plot.

**Dataset:** VertigoSPSSData.sav

**Selected Variable(s):**

**Marker(s):** Zn, Cu, TotalThiol, NativeThiol, Disulfide, ReducedThiol, OxidizedThiol, ThiolOxRedRatio

**Status variable:** Group

**Status:** vertigo

**Show**

**Marker:** Zn

**Descriptive Statistics**

| **Group** | **N** | **Mean** | **Std.Dev.** | **S.E.Mean** | **Mean (Lower)** | **Mean (Upper)** |
| --- | --- | --- | --- | --- | --- | --- |
| Control | 66 | 85.8606 | 7.6114 | 0.9369 | 83.9895 | 87.7317 |
| vertigo | 66 | 87.147 | 13.2657 | 1.6329 | 83.8858 | 90.4081 |

**Test Results & Statistics**

**ROC statistics**

| **AUC** | **S.E.** | **Lower limit** | **Upper limit** | **z value** | **p value** |
| --- | --- | --- | --- | --- | --- |
| 0.5077 | 0.0518 | 0.4193 | 0.5957 | 0.1486 | 0.882 |

When two observations are randomly selected, the test result of an observation with the disease is **50.8%** more likely to be positive than the test result of an observation without the disease.

**ROC coordinates**

| **Cut point** | **FPR** | **TPR** | **FNR** | **TNR** |
| --- | --- | --- | --- | --- |
| -Inf | 0 | 0 | 1 | 1 |
| <=66.3 | 0 | 0.0152 | 0.9848 | 1 |
| <=70.7 | 0 | 0.0303 | 0.9697 | 1 |
| <=73.4 | 0 | 0.0455 | 0.9545 | 1 |
| <=74.6 | 0.0152 | 0.0455 | 0.9545 | 0.9848 |
| <=74.8 | 0.0455 | 0.0455 | 0.9545 | 0.9545 |
| <=74.9 | 0.0606 | 0.0455 | 0.9545 | 0.9394 |
| <=75 | 0.0758 | 0.0455 | 0.9545 | 0.9242 |
| <=75.1 | 0.0909 | 0.0758 | 0.9242 | 0.9091 |
| <=75.3 | 0.1061 | 0.0758 | 0.9242 | 0.8939 |
| <=75.4 | 0.1212 | 0.0758 | 0.9242 | 0.8788 |
| <=75.5 | 0.1364 | 0.0758 | 0.9242 | 0.8636 |
| <=75.9 | 0.1364 | 0.1061 | 0.8939 | 0.8636 |
| <=76 | 0.1515 | 0.1061 | 0.8939 | 0.8485 |
| <=76.1 | 0.1667 | 0.1061 | 0.8939 | 0.8333 |
| <=76.2 | 0.1818 | 0.1061 | 0.8939 | 0.8182 |
| <=76.4 | 0.197 | 0.1061 | 0.8939 | 0.803 |
| <=76.5 | 0.2121 | 0.1061 | 0.8939 | 0.7879 |
| <=76.6 | 0.2273 | 0.1061 | 0.8939 | 0.7727 |
| <=76.7 | 0.2273 | 0.1212 | 0.8788 | 0.7727 |
| <=77 | 0.2273 | 0.1667 | 0.8333 | 0.7727 |
| <=77.3 | 0.2273 | 0.2273 | 0.7727 | 0.7727 |
| <=77.6 | 0.2273 | 0.2424 | 0.7576 | 0.7727 |
| <=78.7 | 0.2273 | 0.2576 | 0.7424 | 0.7727 |
| <=78.9 | 0.2273 | 0.2727 | 0.7273 | 0.7727 |
| <=79.2 | 0.2273 | 0.303 | 0.697 | 0.7727 |
| <=80 | 0.2273 | 0.3182 | 0.6818 | 0.7727 |
| <=80.6 | 0.2273 | 0.3333 | 0.6667 | 0.7727 |
| <=81.1 | 0.2273 | 0.3939 | 0.6061 | 0.7727 |
| <=81.7 | 0.2424 | 0.4242 | 0.5758 | 0.7576 |
| <=81.9 | 0.2576 | 0.4242 | 0.5758 | 0.7424 |
| <=82 | 0.2879 | 0.4394 | 0.5606 | 0.7121 |
| <=82.1 | 0.3182 | 0.4394 | 0.5606 | 0.6818 |
| <=82.2 | 0.3333 | 0.4545 | 0.5455 | 0.6667 |
| <=82.4 | 0.3485 | 0.4545 | 0.5455 | 0.6515 |
| <=82.5 | 0.3636 | 0.4545 | 0.5455 | 0.6364 |
| <=82.6 | 0.3788 | 0.4545 | 0.5455 | 0.6212 |
| <=82.8 | 0.3788 | 0.4848 | 0.5152 | 0.6212 |
| <=83.1 | 0.3788 | 0.5303 | 0.4697 | 0.6212 |
| <=83.6 | 0.3788 | 0.5455 | 0.4545 | 0.6212 |
| <=84.8 | 0.3939 | 0.5455 | 0.4545 | 0.6061 |
| <=84.9 | 0.4091 | 0.5455 | 0.4545 | 0.5909 |
| <=85 | 0.4242 | 0.5455 | 0.4545 | 0.5758 |
| <=85.1 | 0.4394 | 0.5455 | 0.4545 | 0.5606 |
| <=85.2 | 0.4545 | 0.5455 | 0.4545 | 0.5455 |
| <=85.3 | 0.4848 | 0.5455 | 0.4545 | 0.5152 |
| <=85.4 | 0.5 | 0.5455 | 0.4545 | 0.5 |
| <=85.5 | 0.5152 | 0.5455 | 0.4545 | 0.4848 |
| <=86.1 | 0.5152 | 0.5758 | 0.4242 | 0.4848 |
| <=86.4 | 0.5303 | 0.5909 | 0.4091 | 0.4697 |
| <=86.5 | 0.5455 | 0.5909 | 0.4091 | 0.4545 |
| <=86.6 | 0.5606 | 0.5909 | 0.4091 | 0.4394 |
| <=86.8 | 0.5758 | 0.5909 | 0.4091 | 0.4242 |
| <=86.9 | 0.5909 | 0.5909 | 0.4091 | 0.4091 |
| <=87 | 0.6212 | 0.5909 | 0.4091 | 0.3788 |
| <=87.1 | 0.6364 | 0.5909 | 0.4091 | 0.3636 |
| <=87.2 | 0.6515 | 0.6061 | 0.3939 | 0.3485 |
| <=87.3 | 0.6667 | 0.6061 | 0.3939 | 0.3333 |
| <=87.4 | 0.6818 | 0.6061 | 0.3939 | 0.3182 |
| <=87.5 | 0.7121 | 0.6061 | 0.3939 | 0.2879 |
| <=87.6 | 0.7273 | 0.6061 | 0.3939 | 0.2727 |
| <=87.7 | 0.7424 | 0.6061 | 0.3939 | 0.2576 |
| <=88.6 | 0.7424 | 0.6212 | 0.3788 | 0.2576 |
| <=88.8 | 0.7424 | 0.6364 | 0.3636 | 0.2576 |
| <=89.1 | 0.7424 | 0.6515 | 0.3485 | 0.2576 |
| <=89.4 | 0.7424 | 0.7121 | 0.2879 | 0.2576 |
| <=91 | 0.7424 | 0.7273 | 0.2727 | 0.2576 |
| <=91.1 | 0.7424 | 0.7424 | 0.2576 | 0.2576 |
| <=91.3 | 0.7424 | 0.7576 | 0.2424 | 0.2576 |
| <=92.4 | 0.7424 | 0.7727 | 0.2273 | 0.2576 |
| <=93 | 0.7424 | 0.7879 | 0.2121 | 0.2576 |
| <=93.2 | 0.7424 | 0.803 | 0.197 | 0.2576 |
| <=93.5 | 0.7424 | 0.8182 | 0.1818 | 0.2576 |
| <=93.8 | 0.7576 | 0.8182 | 0.1818 | 0.2424 |
| <=93.9 | 0.7727 | 0.8182 | 0.1818 | 0.2273 |
| <=94 | 0.7879 | 0.8182 | 0.1818 | 0.2121 |
| <=94.1 | 0.803 | 0.8182 | 0.1818 | 0.197 |
| <=94.2 | 0.8182 | 0.8182 | 0.1818 | 0.1818 |
| <=94.3 | 0.8485 | 0.8182 | 0.1818 | 0.1515 |
| <=94.4 | 0.8636 | 0.8182 | 0.1818 | 0.1364 |
| <=94.5 | 0.8788 | 0.8182 | 0.1818 | 0.1212 |
| <=94.9 | 0.8788 | 0.8333 | 0.1667 | 0.1212 |
| <=98.2 | 0.8939 | 0.8333 | 0.1667 | 0.1061 |
| <=98.3 | 0.9091 | 0.8333 | 0.1667 | 0.0909 |
| <=98.4 | 0.9242 | 0.8333 | 0.1667 | 0.0758 |
| <=98.5 | 0.9242 | 0.8485 | 0.1515 | 0.0758 |
| <=98.6 | 0.9394 | 0.8485 | 0.1515 | 0.0606 |
| <=99.3 | 0.9545 | 0.8485 | 0.1515 | 0.0455 |
| <=99.5 | 0.9697 | 0.8485 | 0.1515 | 0.0303 |
| <=99.6 | 0.9848 | 0.8485 | 0.1515 | 0.0152 |
| <=99.7 | 1 | 0.8485 | 0.1515 | 0 |
| <=100.4 | 1 | 0.8636 | 0.1364 | 0 |
| <=100.7 | 1 | 0.8788 | 0.1212 | 0 |
| <=100.9 | 1 | 0.8939 | 0.1061 | 0 |
| <=101.2 | 1 | 0.9091 | 0.0909 | 0 |
| <=103.1 | 1 | 0.9242 | 0.0758 | 0 |
| <=107.5 | 1 | 0.9394 | 0.0606 | 0 |
| <=109.7 | 1 | 0.9545 | 0.0455 | 0 |
| <=110.3 | 1 | 0.9697 | 0.0303 | 0 |
| <=122.7 | 1 | 0.9848 | 0.0152 | 0 |
| <=152.4 | 1 | 1 | 0 | 0 |

**Multiple comparisons**

**Test Direction**

**High:** Disulfide, OxidizedThiol

**Low:** Zn, Cu, TotalThiol, NativeThiol, ReducedThiol, ThiolOxRedRatio

| **Marker1 (A)** | **Marker2 (B)** | **AUC (I)** | **AUC (J)** | **Diff. (I,J)** | **S.E. (Diff(A,B))** | **z value** | **p value** | **p value (adj.)** |
| --- | --- | --- | --- | --- | --- | --- | --- | --- |
| Zn | Cu | 0.5077 | 0.6478 | -0.1402 | 0.0431 | -3.2532 | 0.001 | 0.032 |
| Zn | TotalThiol | 0.5077 | 0.7366 | -0.2289 | 0.0466 | -4.91 | <0.001 | <0.001 |
| Zn | NativeThiol | 0.5077 | 0.9403 | -0.4326 | 0.0473 | -9.1465 | <0.001 | <0.001 |
| Zn | Disulfide | 0.5077 | 0.996 | -0.4883 | 0.0498 | -9.8009 | <0.001 | <0.001 |
| Zn | ReducedThiol | 0.5077 | 0.9839 | -0.4762 | 0.0549 | -8.6708 | <0.001 | <0.001 |
| Zn | OxidizedThiol | 0.5077 | 0.9991 | -0.4914 | 0.0517 | -9.4986 | <0.001 | <0.001 |
| Zn | ThiolOxRedRatio | 0.5077 | 0.9986 | -0.4909 | 0.0516 | -9.5156 | <0.001 | <0.001 |
| Cu | TotalThiol | 0.6478 | 0.7366 | -0.0887 | 0.0268 | -3.316 | <0.001 | 0.026 |
| Cu | NativeThiol | 0.6478 | 0.9403 | -0.2925 | 0.0386 | -7.584 | <0.001 | <0.001 |
| Cu | Disulfide | 0.6478 | 0.996 | -0.3481 | 0.0506 | -6.8836 | <0.001 | <0.001 |
| Cu | ReducedThiol | 0.6478 | 0.9839 | -0.3361 | 0.0512 | -6.5646 | <0.001 | <0.001 |
| Cu | OxidizedThiol | 0.6478 | 0.9991 | -0.3512 | 0.046 | -7.6314 | <0.001 | <0.001 |
| Cu | ThiolOxRedRatio | 0.6478 | 0.9986 | -0.3508 | 0.0486 | -7.2211 | <0.001 | <0.001 |
| TotalThiol | NativeThiol | 0.7366 | 0.9403 | -0.2037 | 0.0333 | -6.1134 | <0.001 | <0.001 |
| TotalThiol | Disulfide | 0.7366 | 0.996 | -0.2594 | 0.0447 | -5.7976 | <0.001 | <0.001 |
| TotalThiol | ReducedThiol | 0.7366 | 0.9839 | -0.2474 | 0.0495 | -4.9946 | <0.001 | <0.001 |
| TotalThiol | OxidizedThiol | 0.7366 | 0.9991 | -0.2625 | 0.0441 | -5.9496 | <0.001 | <0.001 |
| TotalThiol | ThiolOxRedRatio | 0.7366 | 0.9986 | -0.2621 | 0.0458 | -5.7165 | <0.001 | <0.001 |
| NativeThiol | Disulfide | 0.9403 | 0.996 | -0.0557 | 0.0207 | -2.6887 | 0.007 | 0.201 |
| NativeThiol | ReducedThiol | 0.9403 | 0.9839 | -0.0436 | 0.0242 | -1.8039 | 0.071 | 1.000 |
| NativeThiol | OxidizedThiol | 0.9403 | 0.9991 | -0.0588 | 0.0195 | -3.0065 | 0.003 | 0.074 |
| NativeThiol | ThiolOxRedRatio | 0.9403 | 0.9986 | -0.0583 | 0.02 | -2.9097 | 0.004 | 0.101 |
| Disulfide | ReducedThiol | 0.996 | 0.9839 | 0.0121 | 0.0149 | 0.8072 | 0.420 | 1.000 |
| Disulfide | OxidizedThiol | 0.996 | 0.9991 | -0.0031 | 0.0023 | -1.3675 | 0.171 | 1.000 |
| Disulfide | ThiolOxRedRatio | 0.996 | 0.9986 | -0.0026 | 0.0026 | -1.0336 | 0.301 | 1.000 |
| ReducedThiol | OxidizedThiol | 0.9839 | 0.9991 | -0.0152 | 0.0153 | -0.9934 | 0.321 | 1.000 |
| ReducedThiol | ThiolOxRedRatio | 0.9839 | 0.9986 | -0.0147 | 0.0147 | -0.9998 | 0.317 | 1.000 |
| OxidizedThiol | ThiolOxRedRatio | 0.9991 | 0.9986 | 0.0005 | 0.0007 | 0.677 | 0.498 | 1.000 |

There is a statistically significant difference between the area under the ROC curves of **Cu** and **Zn** markers. The AUC level of **Cu** marker **(0.6478)** is found to be higher than the AUC level of **Zn** marker **(0.5077)**.

**Cutoff point and diagnostic statistics**

Method: Youden

Criterion: 0.1818

Cutoff: <=81.7

Positive class: vertigo

| **Measures** | **Value** | **Lower limit** | **Upper limit** |
| --- | --- | --- | --- |
| Sensitivity | 0.4242 | 0.3124 | 0.5444 |
| Specificity | 0.7576 | 0.6419 | 0.8449 |
| Positive Predictive Value | 0.6364 | 0.4887 | 0.7622 |
| Negative Predictive Value | 0.5682 | 0.464 | 0.6667 |
| Positive Likelihood Ratio | 1.75 | 1.0501 | 2.9165 |
| Negative Likelihood Ratio | 0.76 | 0.5931 | 0.9739 |

The sensitivity of the **Zn** test is **42.4%**. The probability of the test being positive is **42.4%**, when the disease is present.

The specificity of the **Zn** test is **75.8%**. The probability of the test being negative is **75.8%**, when the disease is not present.

The positive predictive value of the **Zn** test is **63.6%**. The probability of the presence of the disease is **63.6%**, when the test is positive.

The negative predictive value of the **Zn** test is **56.8%**. The probability of the absence of the disease is **56.8%**, when the test is negative.

The positive likelihood ratio of the **Zn** test is **1.750**. The ratio between the probability of a positive test result given the presence of the disease and the probability of a positive test result given the absence of the disease is **1.750**.

The negative likelihood ratio of the **Zn** test is **0.760**. The ratio between the probability of a negative test result given the presence of the disease and the probability of a negative test result given the absence of the disease is **0.760**.

**Show**

**Marker:** Cu

**Descriptive Statistics**

| **Group** | **N** | **Mean** | **Std.Dev.** | **S.E.Mean** | **Mean (Lower)** | **Mean (Upper)** |
| --- | --- | --- | --- | --- | --- | --- |
| Control | 66 | 135.7727 | 42.2603 | 5.2019 | 125.3839 | 146.1616 |
| vertigo | 66 | 119.6061 | 47.632 | 5.8631 | 107.8967 | 131.3155 |

**Test Results & Statistics**

**ROC statistics**

| **AUC** | **S.E.** | **Lower limit** | **Upper limit** | **z value** | **p value** |
| --- | --- | --- | --- | --- | --- |
| 0.6478 | 0.0487 | 0.56 | 0.7289 | 3.0379 | 0.002 |

When two observations are randomly selected, the test result of an observation with the disease is **64.8%** more likely to be positive than the test result of an observation without the disease.

**ROC coordinates**

| **Cut point** | **FPR** | **TPR** | **FNR** | **TNR** |
| --- | --- | --- | --- | --- |
| -Inf | 0 | 0 | 1 | 1 |
| <=58.2 | 0 | 0.0152 | 0.9848 | 1 |
| <=67.1 | 0 | 0.0303 | 0.9697 | 1 |
| <=68.1 | 0 | 0.0455 | 0.9545 | 1 |
| <=72.1 | 0 | 0.0606 | 0.9394 | 1 |
| <=76 | 0 | 0.0758 | 0.9242 | 1 |
| <=77 | 0 | 0.1212 | 0.8788 | 1 |
| <=79 | 0 | 0.1364 | 0.8636 | 1 |
| <=81.9 | 0 | 0.1667 | 0.8333 | 1 |
| <=83.9 | 0 | 0.1818 | 0.8182 | 1 |
| <=84.9 | 0 | 0.197 | 0.803 | 1 |
| <=85.9 | 0 | 0.2121 | 0.7879 | 1 |
| <=86.9 | 0 | 0.2424 | 0.7576 | 1 |
| <=87.8 | 0 | 0.2879 | 0.7121 | 1 |
| <=89.3 | 0.0152 | 0.2879 | 0.7121 | 0.9848 |
| <=89.4 | 0.0303 | 0.2879 | 0.7121 | 0.9697 |
| <=89.5 | 0.0455 | 0.2879 | 0.7121 | 0.9545 |
| <=89.7 | 0.0606 | 0.2879 | 0.7121 | 0.9394 |
| <=89.8 | 0.0758 | 0.3182 | 0.6818 | 0.9242 |
| <=89.9 | 0.0909 | 0.3182 | 0.6818 | 0.9091 |
| <=90.8 | 0.0909 | 0.3333 | 0.6667 | 0.9091 |
| <=91.8 | 0.0909 | 0.3636 | 0.6364 | 0.9091 |
| <=92.8 | 0.0909 | 0.3788 | 0.6212 | 0.9091 |
| <=95.2 | 0.1061 | 0.3788 | 0.6212 | 0.8939 |
| <=95.3 | 0.1212 | 0.3788 | 0.6212 | 0.8788 |
| <=95.4 | 0.1364 | 0.3788 | 0.6212 | 0.8636 |
| <=95.5 | 0.1515 | 0.3788 | 0.6212 | 0.8485 |
| <=95.6 | 0.1667 | 0.3788 | 0.6212 | 0.8333 |
| <=95.7 | 0.197 | 0.3939 | 0.6061 | 0.803 |
| <=95.8 | 0.2121 | 0.3939 | 0.6061 | 0.7879 |
| <=95.9 | 0.2273 | 0.3939 | 0.6061 | 0.7727 |
| <=96.4 | 0.2424 | 0.3939 | 0.6061 | 0.7576 |
| <=96.6 | 0.2576 | 0.3939 | 0.6061 | 0.7424 |
| <=96.7 | 0.2727 | 0.4091 | 0.5909 | 0.7273 |
| <=96.8 | 0.2879 | 0.4091 | 0.5909 | 0.7121 |
| <=97.7 | 0.2879 | 0.4394 | 0.5606 | 0.7121 |
| <=98.7 | 0.2879 | 0.4545 | 0.5455 | 0.7121 |
| <=99.7 | 0.2879 | 0.4697 | 0.5303 | 0.7121 |
| <=100.7 | 0.2879 | 0.5 | 0.5 | 0.7121 |
| <=102.6 | 0.2879 | 0.5152 | 0.4848 | 0.7121 |
| <=103.6 | 0.2879 | 0.5455 | 0.4545 | 0.7121 |
| <=104.6 | 0.2879 | 0.5909 | 0.4091 | 0.7121 |
| <=105.1 | 0.303 | 0.5909 | 0.4091 | 0.697 |
| <=105.2 | 0.3182 | 0.5909 | 0.4091 | 0.6818 |
| <=105.3 | 0.3333 | 0.5909 | 0.4091 | 0.6667 |
| <=105.4 | 0.3485 | 0.5909 | 0.4091 | 0.6515 |
| <=105.5 | 0.3636 | 0.5909 | 0.4091 | 0.6364 |
| <=105.6 | 0.3939 | 0.6061 | 0.3939 | 0.6061 |
| <=105.7 | 0.4091 | 0.6061 | 0.3939 | 0.5909 |
| <=105.8 | 0.4242 | 0.6061 | 0.3939 | 0.5758 |
| <=108.6 | 0.4242 | 0.6212 | 0.3788 | 0.5758 |
| <=109.6 | 0.4242 | 0.6364 | 0.3636 | 0.5758 |
| <=114.5 | 0.4242 | 0.6515 | 0.3485 | 0.5758 |
| <=122.4 | 0.4242 | 0.6667 | 0.3333 | 0.5758 |
| <=127.3 | 0.4242 | 0.6818 | 0.3182 | 0.5758 |
| <=129 | 0.4394 | 0.6818 | 0.3182 | 0.5606 |
| <=129.2 | 0.4545 | 0.6818 | 0.3182 | 0.5455 |
| <=129.3 | 0.4697 | 0.697 | 0.303 | 0.5303 |
| <=129.4 | 0.4848 | 0.697 | 0.303 | 0.5152 |
| <=130.3 | 0.4848 | 0.7121 | 0.2879 | 0.5152 |
| <=132.3 | 0.4848 | 0.7273 | 0.2727 | 0.5152 |
| <=132.7 | 0.5 | 0.7273 | 0.2727 | 0.5 |
| <=132.8 | 0.5152 | 0.7273 | 0.2727 | 0.4848 |
| <=132.9 | 0.5303 | 0.7273 | 0.2727 | 0.4697 |
| <=133.1 | 0.5455 | 0.7273 | 0.2727 | 0.4545 |
| <=133.2 | 0.5606 | 0.7273 | 0.2727 | 0.4394 |
| <=133.3 | 0.5758 | 0.7273 | 0.2727 | 0.4242 |
| <=134.9 | 0.5909 | 0.7273 | 0.2727 | 0.4091 |
| <=135.1 | 0.6061 | 0.7273 | 0.2727 | 0.3939 |
| <=135.2 | 0.6212 | 0.7273 | 0.2727 | 0.3788 |
| <=135.3 | 0.6364 | 0.7273 | 0.2727 | 0.3636 |
| <=139.2 | 0.6364 | 0.7424 | 0.2576 | 0.3636 |
| <=142.6 | 0.6515 | 0.7424 | 0.2576 | 0.3485 |
| <=142.7 | 0.6667 | 0.7424 | 0.2576 | 0.3333 |
| <=142.8 | 0.6818 | 0.7424 | 0.2576 | 0.3182 |
| <=143 | 0.697 | 0.7424 | 0.2576 | 0.303 |
| <=143.1 | 0.7121 | 0.7424 | 0.2576 | 0.2879 |
| <=143.2 | 0.7273 | 0.7424 | 0.2576 | 0.2727 |
| <=144.1 | 0.7273 | 0.7576 | 0.2424 | 0.2727 |
| <=147.1 | 0.7273 | 0.7727 | 0.2273 | 0.2727 |
| <=154 | 0.7273 | 0.803 | 0.197 | 0.2727 |
| <=156.9 | 0.7273 | 0.8182 | 0.1818 | 0.2727 |
| <=157.9 | 0.7273 | 0.8333 | 0.1667 | 0.2727 |
| <=162.9 | 0.7273 | 0.8485 | 0.1515 | 0.2727 |
| <=171.2 | 0.7424 | 0.8485 | 0.1515 | 0.2576 |
| <=171.3 | 0.7576 | 0.8485 | 0.1515 | 0.2424 |
| <=171.4 | 0.7727 | 0.8485 | 0.1515 | 0.2273 |
| <=171.5 | 0.7879 | 0.8485 | 0.1515 | 0.2121 |
| <=171.6 | 0.803 | 0.8485 | 0.1515 | 0.197 |
| <=171.7 | 0.8333 | 0.8485 | 0.1515 | 0.1667 |
| <=171.8 | 0.8485 | 0.8485 | 0.1515 | 0.1515 |
| <=171.9 | 0.8636 | 0.8485 | 0.1515 | 0.1364 |
| <=172.7 | 0.8636 | 0.8636 | 0.1364 | 0.1364 |
| <=183.6 | 0.8636 | 0.8788 | 0.1212 | 0.1364 |
| <=186.5 | 0.8636 | 0.8939 | 0.1061 | 0.1364 |
| <=196.4 | 0.8636 | 0.9091 | 0.0909 | 0.1364 |
| <=211.2 | 0.8636 | 0.9394 | 0.0606 | 0.1364 |
| <=218.6 | 0.8788 | 0.9394 | 0.0606 | 0.1212 |
| <=218.7 | 0.8939 | 0.9394 | 0.0606 | 0.1061 |
| <=218.8 | 0.9091 | 0.9394 | 0.0606 | 0.0909 |
| <=218.9 | 0.9242 | 0.9394 | 0.0606 | 0.0758 |
| <=219 | 0.9394 | 0.9394 | 0.0606 | 0.0606 |
| <=219.1 | 0.9697 | 0.9394 | 0.0606 | 0.0303 |
| <=219.2 | 0.9848 | 0.9394 | 0.0606 | 0.0152 |
| <=219.3 | 1 | 0.9394 | 0.0606 | 0 |
| <=220.1 | 1 | 0.9545 | 0.0455 | 0 |
| <=236.9 | 1 | 0.9697 | 0.0303 | 0 |
| <=255.6 | 1 | 0.9848 | 0.0152 | 0 |
| <=258.6 | 1 | 1 | 0 | 0 |

**Multiple comparisons**

**Test Direction**

**High:** Disulfide, OxidizedThiol

**Low:** Zn, Cu, TotalThiol, NativeThiol, ReducedThiol, ThiolOxRedRatio

| **Marker1 (A)** | **Marker2 (B)** | **AUC (I)** | **AUC (J)** | **Diff. (I,J)** | **S.E. (Diff(A,B))** | **z value** | **p value** | **p value (adj.)** |
| --- | --- | --- | --- | --- | --- | --- | --- | --- |
| Zn | Cu | 0.5077 | 0.6478 | -0.1402 | 0.0431 | -3.2532 | 0.001 | 0.032 |
| Zn | TotalThiol | 0.5077 | 0.7366 | -0.2289 | 0.0466 | -4.91 | <0.001 | <0.001 |
| Zn | NativeThiol | 0.5077 | 0.9403 | -0.4326 | 0.0473 | -9.1465 | <0.001 | <0.001 |
| Zn | Disulfide | 0.5077 | 0.996 | -0.4883 | 0.0498 | -9.8009 | <0.001 | <0.001 |
| Zn | ReducedThiol | 0.5077 | 0.9839 | -0.4762 | 0.0549 | -8.6708 | <0.001 | <0.001 |
| Zn | OxidizedThiol | 0.5077 | 0.9991 | -0.4914 | 0.0517 | -9.4986 | <0.001 | <0.001 |
| Zn | ThiolOxRedRatio | 0.5077 | 0.9986 | -0.4909 | 0.0516 | -9.5156 | <0.001 | <0.001 |
| Cu | TotalThiol | 0.6478 | 0.7366 | -0.0887 | 0.0268 | -3.316 | <0.001 | 0.026 |
| Cu | NativeThiol | 0.6478 | 0.9403 | -0.2925 | 0.0386 | -7.584 | <0.001 | <0.001 |
| Cu | Disulfide | 0.6478 | 0.996 | -0.3481 | 0.0506 | -6.8836 | <0.001 | <0.001 |
| Cu | ReducedThiol | 0.6478 | 0.9839 | -0.3361 | 0.0512 | -6.5646 | <0.001 | <0.001 |
| Cu | OxidizedThiol | 0.6478 | 0.9991 | -0.3512 | 0.046 | -7.6314 | <0.001 | <0.001 |
| Cu | ThiolOxRedRatio | 0.6478 | 0.9986 | -0.3508 | 0.0486 | -7.2211 | <0.001 | <0.001 |
| TotalThiol | NativeThiol | 0.7366 | 0.9403 | -0.2037 | 0.0333 | -6.1134 | <0.001 | <0.001 |
| TotalThiol | Disulfide | 0.7366 | 0.996 | -0.2594 | 0.0447 | -5.7976 | <0.001 | <0.001 |
| TotalThiol | ReducedThiol | 0.7366 | 0.9839 | -0.2474 | 0.0495 | -4.9946 | <0.001 | <0.001 |
| TotalThiol | OxidizedThiol | 0.7366 | 0.9991 | -0.2625 | 0.0441 | -5.9496 | <0.001 | <0.001 |
| TotalThiol | ThiolOxRedRatio | 0.7366 | 0.9986 | -0.2621 | 0.0458 | -5.7165 | <0.001 | <0.001 |
| NativeThiol | Disulfide | 0.9403 | 0.996 | -0.0557 | 0.0207 | -2.6887 | 0.007 | 0.201 |
| NativeThiol | ReducedThiol | 0.9403 | 0.9839 | -0.0436 | 0.0242 | -1.8039 | 0.071 | 1.000 |
| NativeThiol | OxidizedThiol | 0.9403 | 0.9991 | -0.0588 | 0.0195 | -3.0065 | 0.003 | 0.074 |
| NativeThiol | ThiolOxRedRatio | 0.9403 | 0.9986 | -0.0583 | 0.02 | -2.9097 | 0.004 | 0.101 |
| Disulfide | ReducedThiol | 0.996 | 0.9839 | 0.0121 | 0.0149 | 0.8072 | 0.420 | 1.000 |
| Disulfide | OxidizedThiol | 0.996 | 0.9991 | -0.0031 | 0.0023 | -1.3675 | 0.171 | 1.000 |
| Disulfide | ThiolOxRedRatio | 0.996 | 0.9986 | -0.0026 | 0.0026 | -1.0336 | 0.301 | 1.000 |
| ReducedThiol | OxidizedThiol | 0.9839 | 0.9991 | -0.0152 | 0.0153 | -0.9934 | 0.321 | 1.000 |
| ReducedThiol | ThiolOxRedRatio | 0.9839 | 0.9986 | -0.0147 | 0.0147 | -0.9998 | 0.317 | 1.000 |
| OxidizedThiol | ThiolOxRedRatio | 0.9991 | 0.9986 | 0.0005 | 0.0007 | 0.677 | 0.498 | 1.000 |

There is a statistically significant difference between the area under the ROC curves of **Cu** and **Zn** markers. The AUC level of **Cu** marker **(0.6478)** is found to be higher than the AUC level of **Zn** marker **(0.5077)**.

**Cutoff point and diagnostic statistics**

Method: Youden

Criterion: 0.303

Cutoff: <=104.6

Positive class: vertigo

| **Measures** | **Value** | **Lower limit** | **Upper limit** |
| --- | --- | --- | --- |
| Sensitivity | 0.5909 | 0.4705 | 0.7013 |
| Specificity | 0.7121 | 0.5936 | 0.8073 |
| Positive Predictive Value | 0.6724 | 0.5442 | 0.7792 |
| Negative Predictive Value | 0.6351 | 0.5213 | 0.7356 |
| Positive Likelihood Ratio | 2.0526 | 1.3362 | 3.1531 |
| Negative Likelihood Ratio | 0.5745 | 0.4138 | 0.7975 |

The sensitivity of the **Cu** test is **59.1%**. The probability of the test being positive is **59.1%**, when the disease is present.

The specificity of the **Cu** test is **71.2%**. The probability of the test being negative is **71.2%**, when the disease is not present.

The positive predictive value of the **Cu** test is **67.2%**. The probability of the presence of the disease is **67.2%**, when the test is positive.

The negative predictive value of the **Cu** test is **63.5%**. The probability of the absence of the disease is **63.5%**, when the test is negative.

The positive likelihood ratio of the **Cu** test is **2.053**. The ratio between the probability of a positive test result given the presence of the disease and the probability of a positive test result given the absence of the disease is **2.053**.

The negative likelihood ratio of the **Cu** test is **0.575**. The ratio between the probability of a negative test result given the presence of the disease and the probability of a negative test result given the absence of the disease is **0.575**.

**Show**

**Marker:** TotalThiol

**Descriptive Statistics**

| **Group** | **N** | **Mean** | **Std.Dev.** | **S.E.Mean** | **Mean (Lower)** | **Mean (Upper)** |
| --- | --- | --- | --- | --- | --- | --- |
| Control | 66 | 611.7333 | 84.8465 | 10.4439 | 590.8755 | 632.5912 |
| vertigo | 66 | 535.0273 | 154.7183 | 19.0445 | 496.9928 | 573.0618 |

**Test Results & Statistics**

**ROC statistics**

| **AUC** | **S.E.** | **Lower limit** | **Upper limit** | **z value** | **p value** |
| --- | --- | --- | --- | --- | --- |
| 0.7366 | 0.0458 | 0.6528 | 0.8094 | 5.1603 | <0.001 |

When two observations are randomly selected, the test result of an observation with the disease is **73.7%** more likely to be positive than the test result of an observation without the disease.

**ROC coordinates**

| **Cut point** | **FPR** | **TPR** | **FNR** | **TNR** |
| --- | --- | --- | --- | --- |
| -Inf | 0 | 0 | 1 | 1 |
| <=378 | 0 | 0.0303 | 0.9697 | 1 |
| <=385.8 | 0 | 0.0455 | 0.9545 | 1 |
| <=387.3 | 0 | 0.0606 | 0.9394 | 1 |
| <=396.6 | 0 | 0.0758 | 0.9242 | 1 |
| <=399.6 | 0 | 0.0909 | 0.9091 | 1 |
| <=402.7 | 0 | 0.1061 | 0.8939 | 1 |
| <=404.3 | 0 | 0.1212 | 0.8788 | 1 |
| <=405.8 | 0 | 0.1364 | 0.8636 | 1 |
| <=407.4 | 0 | 0.1515 | 0.8485 | 1 |
| <=408.9 | 0 | 0.1667 | 0.8333 | 1 |
| <=410.4 | 0 | 0.1818 | 0.8182 | 1 |
| <=412 | 0 | 0.197 | 0.803 | 1 |
| <=415.1 | 0 | 0.2121 | 0.7879 | 1 |
| <=419.7 | 0 | 0.2273 | 0.7727 | 1 |
| <=421.2 | 0 | 0.2424 | 0.7576 | 1 |
| <=422.8 | 0 | 0.2576 | 0.7424 | 1 |
| <=424.3 | 0 | 0.2727 | 0.7273 | 1 |
| <=433.6 | 0 | 0.2879 | 0.7121 | 1 |
| <=438.2 | 0 | 0.303 | 0.697 | 1 |
| <=439.8 | 0 | 0.3182 | 0.6818 | 1 |
| <=444.4 | 0 | 0.3333 | 0.6667 | 1 |
| <=445.9 | 0 | 0.3485 | 0.6515 | 1 |
| <=447.5 | 0 | 0.3636 | 0.6364 | 1 |
| <=449 | 0 | 0.3788 | 0.6212 | 1 |
| <=452.1 | 0 | 0.3939 | 0.6061 | 1 |
| <=458.3 | 0 | 0.4091 | 0.5909 | 1 |
| <=459.8 | 0 | 0.4242 | 0.5758 | 1 |
| <=462.9 | 0 | 0.4394 | 0.5606 | 1 |
| <=470.6 | 0 | 0.4545 | 0.5455 | 1 |
| <=478.3 | 0 | 0.4697 | 0.5303 | 1 |
| <=482.7 | 0.0152 | 0.4697 | 0.5303 | 0.9848 |
| <=482.9 | 0.0303 | 0.4697 | 0.5303 | 0.9697 |
| <=483 | 0.0455 | 0.4697 | 0.5303 | 0.9545 |
| <=483.1 | 0.0606 | 0.4697 | 0.5303 | 0.9394 |
| <=485.5 | 0.0758 | 0.4697 | 0.5303 | 0.9242 |
| <=485.6 | 0.0909 | 0.4697 | 0.5303 | 0.9091 |
| <=485.7 | 0.1061 | 0.4697 | 0.5303 | 0.8939 |
| <=485.8 | 0.1212 | 0.4697 | 0.5303 | 0.8788 |
| <=485.9 | 0.1364 | 0.4697 | 0.5303 | 0.8636 |
| <=486 | 0.1667 | 0.4848 | 0.5152 | 0.8333 |
| <=486.1 | 0.1818 | 0.4848 | 0.5152 | 0.8182 |
| <=486.2 | 0.197 | 0.4848 | 0.5152 | 0.803 |
| <=495.3 | 0.197 | 0.5152 | 0.4848 | 0.803 |
| <=496.8 | 0.197 | 0.5303 | 0.4697 | 0.803 |
| <=507.6 | 0.197 | 0.5455 | 0.4545 | 0.803 |
| <=509.2 | 0.197 | 0.5606 | 0.4394 | 0.803 |
| <=513.8 | 0.197 | 0.5758 | 0.4242 | 0.803 |
| <=523.1 | 0.197 | 0.6061 | 0.3939 | 0.803 |
| <=524.6 | 0.197 | 0.6212 | 0.3788 | 0.803 |
| <=537 | 0.197 | 0.6364 | 0.3636 | 0.803 |
| <=546.2 | 0.197 | 0.6515 | 0.3485 | 0.803 |
| <=547.8 | 0.197 | 0.6667 | 0.3333 | 0.803 |
| <=550.9 | 0.197 | 0.697 | 0.303 | 0.803 |
| <=552.4 | 0.197 | 0.7121 | 0.2879 | 0.803 |
| <=570.4 | 0.2121 | 0.7121 | 0.2879 | 0.7879 |
| <=570.5 | 0.2273 | 0.7121 | 0.2879 | 0.7727 |
| <=570.6 | 0.2424 | 0.7121 | 0.2879 | 0.7576 |
| <=570.8 | 0.2576 | 0.7121 | 0.2879 | 0.7424 |
| <=570.9 | 0.2727 | 0.7121 | 0.2879 | 0.7273 |
| <=571 | 0.2879 | 0.7121 | 0.2879 | 0.7121 |
| <=572.5 | 0.2879 | 0.7273 | 0.2727 | 0.7121 |
| <=585.8 | 0.303 | 0.7273 | 0.2727 | 0.697 |
| <=585.9 | 0.3182 | 0.7273 | 0.2727 | 0.6818 |
| <=586 | 0.3333 | 0.7273 | 0.2727 | 0.6667 |
| <=586.1 | 0.3485 | 0.7273 | 0.2727 | 0.6515 |
| <=586.2 | 0.3636 | 0.7273 | 0.2727 | 0.6364 |
| <=586.3 | 0.3939 | 0.7273 | 0.2727 | 0.6061 |
| <=586.4 | 0.4091 | 0.7273 | 0.2727 | 0.5909 |
| <=586.5 | 0.4242 | 0.7273 | 0.2727 | 0.5758 |
| <=589.4 | 0.4242 | 0.7424 | 0.2576 | 0.5758 |
| <=593.6 | 0.4394 | 0.7424 | 0.2576 | 0.5606 |
| <=593.7 | 0.4545 | 0.7424 | 0.2576 | 0.5455 |
| <=593.8 | 0.4697 | 0.7424 | 0.2576 | 0.5303 |
| <=594 | 0.4848 | 0.7424 | 0.2576 | 0.5152 |
| <=594.1 | 0.5 | 0.7424 | 0.2576 | 0.5 |
| <=594.2 | 0.5152 | 0.7424 | 0.2576 | 0.4848 |
| <=601.5 | 0.5303 | 0.7424 | 0.2576 | 0.4697 |
| <=601.7 | 0.5455 | 0.7424 | 0.2576 | 0.4545 |
| <=601.8 | 0.5606 | 0.7576 | 0.2424 | 0.4394 |
| <=601.9 | 0.5758 | 0.7576 | 0.2424 | 0.4242 |
| <=604.9 | 0.5758 | 0.7879 | 0.2121 | 0.4242 |
| <=627.7 | 0.5909 | 0.7879 | 0.2121 | 0.4091 |
| <=627.9 | 0.6061 | 0.7879 | 0.2121 | 0.3939 |
| <=628 | 0.6212 | 0.7879 | 0.2121 | 0.3788 |
| <=628.1 | 0.6364 | 0.7879 | 0.2121 | 0.3636 |
| <=629.5 | 0.6364 | 0.803 | 0.197 | 0.3636 |
| <=636.8 | 0.6515 | 0.803 | 0.197 | 0.3485 |
| <=636.9 | 0.6667 | 0.803 | 0.197 | 0.3333 |
| <=637 | 0.6818 | 0.803 | 0.197 | 0.3182 |
| <=637.2 | 0.697 | 0.803 | 0.197 | 0.303 |
| <=637.3 | 0.7121 | 0.803 | 0.197 | 0.2879 |
| <=637.4 | 0.7273 | 0.803 | 0.197 | 0.2727 |
| <=649.6 | 0.7273 | 0.8182 | 0.1818 | 0.2727 |
| <=660.4 | 0.7273 | 0.8333 | 0.1667 | 0.2727 |
| <=665 | 0.7273 | 0.8485 | 0.1515 | 0.2727 |
| <=669.7 | 0.7273 | 0.8636 | 0.1364 | 0.2727 |
| <=694.4 | 0.7273 | 0.8788 | 0.1212 | 0.2727 |
| <=706.7 | 0.7273 | 0.8939 | 0.1061 | 0.2727 |
| <=717 | 0.7424 | 0.8939 | 0.1061 | 0.2576 |
| <=717.1 | 0.7576 | 0.8939 | 0.1061 | 0.2424 |
| <=717.2 | 0.7727 | 0.8939 | 0.1061 | 0.2273 |
| <=717.3 | 0.7879 | 0.8939 | 0.1061 | 0.2121 |
| <=717.4 | 0.803 | 0.8939 | 0.1061 | 0.197 |
| <=717.5 | 0.8333 | 0.8939 | 0.1061 | 0.1667 |
| <=717.6 | 0.8485 | 0.8939 | 0.1061 | 0.1515 |
| <=717.7 | 0.8636 | 0.8939 | 0.1061 | 0.1364 |
| <=722.1 | 0.8636 | 0.9091 | 0.0909 | 0.1364 |
| <=734 | 0.8788 | 0.9091 | 0.0909 | 0.1212 |
| <=734.1 | 0.8939 | 0.9091 | 0.0909 | 0.1061 |
| <=734.2 | 0.9091 | 0.9091 | 0.0909 | 0.0909 |
| <=734.3 | 0.9242 | 0.9091 | 0.0909 | 0.0758 |
| <=734.4 | 0.9394 | 0.9091 | 0.0909 | 0.0606 |
| <=734.5 | 0.9697 | 0.9091 | 0.0909 | 0.0303 |
| <=734.6 | 0.9848 | 0.9091 | 0.0909 | 0.0152 |
| <=734.7 | 1 | 0.9091 | 0.0909 | 0 |
| <=737.6 | 1 | 0.9242 | 0.0758 | 0 |
| <=765.3 | 1 | 0.9394 | 0.0606 | 0 |
| <=813.2 | 1 | 0.9545 | 0.0455 | 0 |
| <=919.6 | 1 | 0.9697 | 0.0303 | 0 |
| <=987.5 | 1 | 0.9848 | 0.0152 | 0 |
| <=1197.4 | 1 | 1 | 0 | 0 |

**Multiple comparisons**

**Test Direction**

**High:** Disulfide, OxidizedThiol

**Low:** Zn, Cu, TotalThiol, NativeThiol, ReducedThiol, ThiolOxRedRatio

| **Marker1 (A)** | **Marker2 (B)** | **AUC (I)** | **AUC (J)** | **Diff. (I,J)** | **S.E. (Diff(A,B))** | **z value** | **p value** | **p value (adj.)** |
| --- | --- | --- | --- | --- | --- | --- | --- | --- |
| Zn | Cu | 0.5077 | 0.6478 | -0.1402 | 0.0431 | -3.2532 | 0.001 | 0.032 |
| Zn | TotalThiol | 0.5077 | 0.7366 | -0.2289 | 0.0466 | -4.91 | <0.001 | <0.001 |
| Zn | NativeThiol | 0.5077 | 0.9403 | -0.4326 | 0.0473 | -9.1465 | <0.001 | <0.001 |
| Zn | Disulfide | 0.5077 | 0.996 | -0.4883 | 0.0498 | -9.8009 | <0.001 | <0.001 |
| Zn | ReducedThiol | 0.5077 | 0.9839 | -0.4762 | 0.0549 | -8.6708 | <0.001 | <0.001 |
| Zn | OxidizedThiol | 0.5077 | 0.9991 | -0.4914 | 0.0517 | -9.4986 | <0.001 | <0.001 |
| Zn | ThiolOxRedRatio | 0.5077 | 0.9986 | -0.4909 | 0.0516 | -9.5156 | <0.001 | <0.001 |
| Cu | TotalThiol | 0.6478 | 0.7366 | -0.0887 | 0.0268 | -3.316 | <0.001 | 0.026 |
| Cu | NativeThiol | 0.6478 | 0.9403 | -0.2925 | 0.0386 | -7.584 | <0.001 | <0.001 |
| Cu | Disulfide | 0.6478 | 0.996 | -0.3481 | 0.0506 | -6.8836 | <0.001 | <0.001 |
| Cu | ReducedThiol | 0.6478 | 0.9839 | -0.3361 | 0.0512 | -6.5646 | <0.001 | <0.001 |
| Cu | OxidizedThiol | 0.6478 | 0.9991 | -0.3512 | 0.046 | -7.6314 | <0.001 | <0.001 |
| Cu | ThiolOxRedRatio | 0.6478 | 0.9986 | -0.3508 | 0.0486 | -7.2211 | <0.001 | <0.001 |
| TotalThiol | NativeThiol | 0.7366 | 0.9403 | -0.2037 | 0.0333 | -6.1134 | <0.001 | <0.001 |
| TotalThiol | Disulfide | 0.7366 | 0.996 | -0.2594 | 0.0447 | -5.7976 | <0.001 | <0.001 |
| TotalThiol | ReducedThiol | 0.7366 | 0.9839 | -0.2474 | 0.0495 | -4.9946 | <0.001 | <0.001 |
| TotalThiol | OxidizedThiol | 0.7366 | 0.9991 | -0.2625 | 0.0441 | -5.9496 | <0.001 | <0.001 |
| TotalThiol | ThiolOxRedRatio | 0.7366 | 0.9986 | -0.2621 | 0.0458 | -5.7165 | <0.001 | <0.001 |
| NativeThiol | Disulfide | 0.9403 | 0.996 | -0.0557 | 0.0207 | -2.6887 | 0.007 | 0.201 |
| NativeThiol | ReducedThiol | 0.9403 | 0.9839 | -0.0436 | 0.0242 | -1.8039 | 0.071 | 1.000 |
| NativeThiol | OxidizedThiol | 0.9403 | 0.9991 | -0.0588 | 0.0195 | -3.0065 | 0.003 | 0.074 |
| NativeThiol | ThiolOxRedRatio | 0.9403 | 0.9986 | -0.0583 | 0.02 | -2.9097 | 0.004 | 0.101 |
| Disulfide | ReducedThiol | 0.996 | 0.9839 | 0.0121 | 0.0149 | 0.8072 | 0.420 | 1.000 |
| Disulfide | OxidizedThiol | 0.996 | 0.9991 | -0.0031 | 0.0023 | -1.3675 | 0.171 | 1.000 |
| Disulfide | ThiolOxRedRatio | 0.996 | 0.9986 | -0.0026 | 0.0026 | -1.0336 | 0.301 | 1.000 |
| ReducedThiol | OxidizedThiol | 0.9839 | 0.9991 | -0.0152 | 0.0153 | -0.9934 | 0.321 | 1.000 |
| ReducedThiol | ThiolOxRedRatio | 0.9839 | 0.9986 | -0.0147 | 0.0147 | -0.9998 | 0.317 | 1.000 |
| OxidizedThiol | ThiolOxRedRatio | 0.9991 | 0.9986 | 0.0005 | 0.0007 | 0.677 | 0.498 | 1.000 |

There is a statistically significant difference between the area under the ROC curves of **Cu** and **Zn** markers. The AUC level of **Cu** marker **(0.6478)** is found to be higher than the AUC level of **Zn** marker **(0.5077)**.

**Cutoff point and diagnostic statistics**

Method: Youden

Criterion: 0.5152

Cutoff: <=552.4

Positive class: vertigo

| **Measures** | **Value** | **Lower limit** | **Upper limit** |
| --- | --- | --- | --- |
| Sensitivity | 0.7121 | 0.5936 | 0.8073 |
| Specificity | 0.803 | 0.6916 | 0.8811 |
| Positive Predictive Value | 0.7833 | 0.6638 | 0.8688 |
| Negative Predictive Value | 0.7361 | 0.6242 | 0.8241 |
| Positive Likelihood Ratio | 3.6154 | 2.1695 | 6.0249 |
| Negative Likelihood Ratio | 0.3585 | 0.2408 | 0.5336 |

The sensitivity of the **TotalThiol** test is **71.2%**. The probability of the test being positive is **71.2%**, when the disease is present.

The specificity of the **TotalThiol** test is **80.3%**. The probability of the test being negative is **80.3%**, when the disease is not present.

The positive predictive value of the **TotalThiol** test is **78.3%**. The probability of the presence of the disease is **78.3%**, when the test is positive.

The negative predictive value of the **TotalThiol** test is **73.6%**. The probability of the absence of the disease is **73.6%**, when the test is negative.

The positive likelihood ratio of the **TotalThiol** test is **3.615**. The ratio between the probability of a positive test result given the presence of the disease and the probability of a positive test result given the absence of the disease is **3.615**.

The negative likelihood ratio of the **TotalThiol** test is **0.358**. The ratio between the probability of a negative test result given the presence of the disease and the probability of a negative test result given the absence of the disease is **0.358**.

**Show**

**Marker:** NativeThiol

**Descriptive Statistics**

| **Group** | **N** | **Mean** | **Std.Dev.** | **S.E.Mean** | **Mean (Lower)** | **Mean (Upper)** |
| --- | --- | --- | --- | --- | --- | --- |
| Control | 66 | 551.2788 | 89.3462 | 10.9978 | 529.3147 | 573.2428 |
| vertigo | 66 | 328.497 | 98.1599 | 12.0826 | 304.3663 | 352.6277 |

**Test Results & Statistics**

**ROC statistics**

| **AUC** | **S.E.** | **Lower limit** | **Upper limit** | **z value** | **p value** |
| --- | --- | --- | --- | --- | --- |
| 0.9403 | 0.0202 | 0.8852 | 0.9741 | 21.82 | <0.001 |

When two observations are randomly selected, the test result of an observation with the disease is **94%** more likely to be positive than the test result of an observation without the disease.

**ROC coordinates**

| **Cut point** | **FPR** | **TPR** | **FNR** | **TNR** |
| --- | --- | --- | --- | --- |
| -Inf | 0 | 0 | 1 | 1 |
| <=162.9 | 0 | 0.0152 | 0.9848 | 1 |
| <=167.8 | 0 | 0.0303 | 0.9697 | 1 |
| <=215.2 | 0 | 0.0455 | 0.9545 | 1 |
| <=224 | 0 | 0.0606 | 0.9394 | 1 |
| <=231 | 0 | 0.0758 | 0.9242 | 1 |
| <=234.9 | 0 | 0.1061 | 0.8939 | 1 |
| <=237.9 | 0 | 0.1212 | 0.8788 | 1 |
| <=239.8 | 0 | 0.1364 | 0.8636 | 1 |
| <=241.8 | 0 | 0.1667 | 0.8333 | 1 |
| <=248.7 | 0 | 0.1818 | 0.8182 | 1 |
| <=251.7 | 0 | 0.197 | 0.803 | 1 |
| <=258.6 | 0 | 0.2121 | 0.7879 | 1 |
| <=263.5 | 0 | 0.2273 | 0.7727 | 1 |
| <=264.5 | 0 | 0.2727 | 0.7273 | 1 |
| <=268.5 | 0 | 0.2879 | 0.7121 | 1 |
| <=269.5 | 0 | 0.303 | 0.697 | 1 |
| <=270.4 | 0 | 0.3485 | 0.6515 | 1 |
| <=276.4 | 0 | 0.3636 | 0.6364 | 1 |
| <=280.3 | 0 | 0.3939 | 0.6061 | 1 |
| <=282.3 | 0 | 0.4091 | 0.5909 | 1 |
| <=285.2 | 0 | 0.4394 | 0.5606 | 1 |
| <=291.2 | 0 | 0.4697 | 0.5303 | 1 |
| <=293.1 | 0 | 0.4848 | 0.5152 | 1 |
| <=294.1 | 0 | 0.5 | 0.5 | 1 |
| <=296.1 | 0 | 0.5152 | 0.4848 | 1 |
| <=298.1 | 0 | 0.5303 | 0.4697 | 1 |
| <=301 | 0 | 0.5455 | 0.4545 | 1 |
| <=311.9 | 0 | 0.5606 | 0.4394 | 1 |
| <=321.8 | 0 | 0.5758 | 0.4242 | 1 |
| <=322.7 | 0 | 0.5909 | 0.4091 | 1 |
| <=328.7 | 0 | 0.6061 | 0.3939 | 1 |
| <=329.7 | 0 | 0.6212 | 0.3788 | 1 |
| <=332.6 | 0 | 0.6364 | 0.3636 | 1 |
| <=342.5 | 0 | 0.6515 | 0.3485 | 1 |
| <=344.5 | 0 | 0.6818 | 0.3182 | 1 |
| <=348.4 | 0 | 0.697 | 0.303 | 1 |
| <=356.3 | 0 | 0.7121 | 0.2879 | 1 |
| <=357.3 | 0 | 0.7273 | 0.2727 | 1 |
| <=365.2 | 0 | 0.7424 | 0.2576 | 1 |
| <=366.9 | 0.0152 | 0.7424 | 0.2576 | 0.9848 |
| <=367 | 0.0303 | 0.7424 | 0.2576 | 0.9697 |
| <=375.1 | 0.0303 | 0.7576 | 0.2424 | 0.9697 |
| <=381 | 0.0303 | 0.7727 | 0.2273 | 0.9697 |
| <=383 | 0.0303 | 0.7879 | 0.2121 | 0.9697 |
| <=400.6 | 0.0455 | 0.7879 | 0.2121 | 0.9545 |
| <=400.7 | 0.0455 | 0.803 | 0.197 | 0.9545 |
| <=416.5 | 0.0455 | 0.8182 | 0.1818 | 0.9545 |
| <=421.2 | 0.0606 | 0.8182 | 0.1818 | 0.9394 |
| <=421.4 | 0.0758 | 0.8182 | 0.1818 | 0.9242 |
| <=421.6 | 0.0909 | 0.8182 | 0.1818 | 0.9091 |
| <=422.8 | 0.1061 | 0.8182 | 0.1818 | 0.8939 |
| <=426.5 | 0.1212 | 0.8182 | 0.1818 | 0.8788 |
| <=426.6 | 0.1364 | 0.8182 | 0.1818 | 0.8636 |
| <=426.7 | 0.1515 | 0.8182 | 0.1818 | 0.8485 |
| <=426.9 | 0.1667 | 0.8182 | 0.1818 | 0.8333 |
| <=434.3 | 0.1667 | 0.8333 | 0.1667 | 0.8333 |
| <=436.3 | 0.1667 | 0.8485 | 0.1515 | 0.8333 |
| <=445.1 | 0.1818 | 0.8485 | 0.1515 | 0.8182 |
| <=445.3 | 0.197 | 0.8485 | 0.1515 | 0.803 |
| <=446.1 | 0.197 | 0.8636 | 0.1364 | 0.803 |
| <=455 | 0.197 | 0.8788 | 0.1212 | 0.803 |
| <=459 | 0.197 | 0.8939 | 0.1061 | 0.803 |
| <=466.9 | 0.197 | 0.9091 | 0.0909 | 0.803 |
| <=469.2 | 0.2121 | 0.9091 | 0.0909 | 0.7879 |
| <=469.3 | 0.2273 | 0.9091 | 0.0909 | 0.7727 |
| <=471.8 | 0.2273 | 0.9242 | 0.0758 | 0.7727 |
| <=486.9 | 0.2424 | 0.9242 | 0.0758 | 0.7576 |
| <=487.2 | 0.2576 | 0.9242 | 0.0758 | 0.7424 |
| <=487.3 | 0.2727 | 0.9242 | 0.0758 | 0.7273 |
| <=496.3 | 0.2879 | 0.9242 | 0.0758 | 0.7121 |
| <=500.2 | 0.303 | 0.9242 | 0.0758 | 0.697 |
| <=502.4 | 0.303 | 0.9394 | 0.0606 | 0.697 |
| <=510.3 | 0.303 | 0.9545 | 0.0455 | 0.697 |
| <=521.4 | 0.3182 | 0.9545 | 0.0455 | 0.6818 |
| <=522.3 | 0.3333 | 0.9545 | 0.0455 | 0.6667 |
| <=534 | 0.3333 | 0.9697 | 0.0303 | 0.6667 |
| <=542.1 | 0.3485 | 0.9697 | 0.0303 | 0.6515 |
| <=542.3 | 0.3636 | 0.9697 | 0.0303 | 0.6364 |
| <=545.3 | 0.3788 | 0.9697 | 0.0303 | 0.6212 |
| <=545.9 | 0.3939 | 0.9697 | 0.0303 | 0.6061 |
| <=546 | 0.4091 | 0.9697 | 0.0303 | 0.5909 |
| <=546.1 | 0.4242 | 0.9697 | 0.0303 | 0.5758 |
| <=546.3 | 0.4545 | 0.9697 | 0.0303 | 0.5455 |
| <=546.9 | 0.4697 | 0.9697 | 0.0303 | 0.5303 |
| <=549.2 | 0.4848 | 0.9697 | 0.0303 | 0.5152 |
| <=549.3 | 0.5152 | 0.9697 | 0.0303 | 0.4848 |
| <=549.4 | 0.5303 | 0.9697 | 0.0303 | 0.4697 |
| <=549.6 | 0.5455 | 0.9697 | 0.0303 | 0.4545 |
| <=559 | 0.5606 | 0.9697 | 0.0303 | 0.4394 |
| <=559.1 | 0.5909 | 0.9697 | 0.0303 | 0.4091 |
| <=559.2 | 0.6061 | 0.9697 | 0.0303 | 0.3939 |
| <=559.3 | 0.6212 | 0.9697 | 0.0303 | 0.3788 |
| <=588 | 0.6364 | 0.9697 | 0.0303 | 0.3636 |
| <=588.2 | 0.6515 | 0.9697 | 0.0303 | 0.3485 |
| <=588.3 | 0.6515 | 0.9848 | 0.0152 | 0.3485 |
| <=596.2 | 0.6667 | 0.9848 | 0.0152 | 0.3333 |
| <=599.7 | 0.6818 | 0.9848 | 0.0152 | 0.3182 |
| <=599.8 | 0.697 | 0.9848 | 0.0152 | 0.303 |
| <=599.9 | 0.7121 | 0.9848 | 0.0152 | 0.2879 |
| <=600.1 | 0.7273 | 0.9848 | 0.0152 | 0.2727 |
| <=600.3 | 0.7424 | 0.9848 | 0.0152 | 0.2576 |
| <=621.3 | 0.7576 | 0.9848 | 0.0152 | 0.2424 |
| <=629.3 | 0.7727 | 0.9848 | 0.0152 | 0.2273 |
| <=647.9 | 0.7879 | 0.9848 | 0.0152 | 0.2121 |
| <=648.1 | 0.803 | 0.9848 | 0.0152 | 0.197 |
| <=648.3 | 0.8182 | 0.9848 | 0.0152 | 0.1818 |
| <=662.3 | 0.8182 | 1 | 0 | 0.1818 |
| <=668.8 | 0.8333 | 1 | 0 | 0.1667 |
| <=668.9 | 0.8636 | 1 | 0 | 0.1364 |
| <=669 | 0.8788 | 1 | 0 | 0.1212 |
| <=669.1 | 0.8939 | 1 | 0 | 0.1061 |
| <=669.2 | 0.9091 | 1 | 0 | 0.0909 |
| <=669.3 | 0.9242 | 1 | 0 | 0.0758 |
| <=669.4 | 0.9394 | 1 | 0 | 0.0606 |
| <=688.9 | 0.9545 | 1 | 0 | 0.0455 |
| <=689 | 0.9697 | 1 | 0 | 0.0303 |
| <=689.1 | 0.9848 | 1 | 0 | 0.0152 |
| <=689.3 | 1 | 1 | 0 | 0 |

**Multiple comparisons**

**Test Direction**

**High:** Disulfide, OxidizedThiol

**Low:** Zn, Cu, TotalThiol, NativeThiol, ReducedThiol, ThiolOxRedRatio

| **Marker1 (A)** | **Marker2 (B)** | **AUC (I)** | **AUC (J)** | **Diff. (I,J)** | **S.E. (Diff(A,B))** | **z value** | **p value** | **p value (adj.)** |
| --- | --- | --- | --- | --- | --- | --- | --- | --- |
| Zn | Cu | 0.5077 | 0.6478 | -0.1402 | 0.0431 | -3.2532 | 0.001 | 0.032 |
| Zn | TotalThiol | 0.5077 | 0.7366 | -0.2289 | 0.0466 | -4.91 | <0.001 | <0.001 |
| Zn | NativeThiol | 0.5077 | 0.9403 | -0.4326 | 0.0473 | -9.1465 | <0.001 | <0.001 |
| Zn | Disulfide | 0.5077 | 0.996 | -0.4883 | 0.0498 | -9.8009 | <0.001 | <0.001 |
| Zn | ReducedThiol | 0.5077 | 0.9839 | -0.4762 | 0.0549 | -8.6708 | <0.001 | <0.001 |
| Zn | OxidizedThiol | 0.5077 | 0.9991 | -0.4914 | 0.0517 | -9.4986 | <0.001 | <0.001 |
| Zn | ThiolOxRedRatio | 0.5077 | 0.9986 | -0.4909 | 0.0516 | -9.5156 | <0.001 | <0.001 |
| Cu | TotalThiol | 0.6478 | 0.7366 | -0.0887 | 0.0268 | -3.316 | <0.001 | 0.026 |
| Cu | NativeThiol | 0.6478 | 0.9403 | -0.2925 | 0.0386 | -7.584 | <0.001 | <0.001 |
| Cu | Disulfide | 0.6478 | 0.996 | -0.3481 | 0.0506 | -6.8836 | <0.001 | <0.001 |
| Cu | ReducedThiol | 0.6478 | 0.9839 | -0.3361 | 0.0512 | -6.5646 | <0.001 | <0.001 |
| Cu | OxidizedThiol | 0.6478 | 0.9991 | -0.3512 | 0.046 | -7.6314 | <0.001 | <0.001 |
| Cu | ThiolOxRedRatio | 0.6478 | 0.9986 | -0.3508 | 0.0486 | -7.2211 | <0.001 | <0.001 |
| TotalThiol | NativeThiol | 0.7366 | 0.9403 | -0.2037 | 0.0333 | -6.1134 | <0.001 | <0.001 |
| TotalThiol | Disulfide | 0.7366 | 0.996 | -0.2594 | 0.0447 | -5.7976 | <0.001 | <0.001 |
| TotalThiol | ReducedThiol | 0.7366 | 0.9839 | -0.2474 | 0.0495 | -4.9946 | <0.001 | <0.001 |
| TotalThiol | OxidizedThiol | 0.7366 | 0.9991 | -0.2625 | 0.0441 | -5.9496 | <0.001 | <0.001 |
| TotalThiol | ThiolOxRedRatio | 0.7366 | 0.9986 | -0.2621 | 0.0458 | -5.7165 | <0.001 | <0.001 |
| NativeThiol | Disulfide | 0.9403 | 0.996 | -0.0557 | 0.0207 | -2.6887 | 0.007 | 0.201 |
| NativeThiol | ReducedThiol | 0.9403 | 0.9839 | -0.0436 | 0.0242 | -1.8039 | 0.071 | 1.000 |
| NativeThiol | OxidizedThiol | 0.9403 | 0.9991 | -0.0588 | 0.0195 | -3.0065 | 0.003 | 0.074 |
| NativeThiol | ThiolOxRedRatio | 0.9403 | 0.9986 | -0.0583 | 0.02 | -2.9097 | 0.004 | 0.101 |
| Disulfide | ReducedThiol | 0.996 | 0.9839 | 0.0121 | 0.0149 | 0.8072 | 0.420 | 1.000 |
| Disulfide | OxidizedThiol | 0.996 | 0.9991 | -0.0031 | 0.0023 | -1.3675 | 0.171 | 1.000 |
| Disulfide | ThiolOxRedRatio | 0.996 | 0.9986 | -0.0026 | 0.0026 | -1.0336 | 0.301 | 1.000 |
| ReducedThiol | OxidizedThiol | 0.9839 | 0.9991 | -0.0152 | 0.0153 | -0.9934 | 0.321 | 1.000 |
| ReducedThiol | ThiolOxRedRatio | 0.9839 | 0.9986 | -0.0147 | 0.0147 | -0.9998 | 0.317 | 1.000 |
| OxidizedThiol | ThiolOxRedRatio | 0.9991 | 0.9986 | 0.0005 | 0.0007 | 0.677 | 0.498 | 1.000 |

There is a statistically significant difference between the area under the ROC curves of **Cu** and **Zn** markers. The AUC level of **Cu** marker **(0.6478)** is found to be higher than the AUC level of **Zn** marker **(0.5077)**.

**Cutoff point and diagnostic statistics**

Method: Youden

Criterion: 0.7727

Cutoff: <=416.5

Positive class: vertigo

| **Measures** | **Value** | **Lower limit** | **Upper limit** |
| --- | --- | --- | --- |
| Sensitivity | 0.8182 | 0.7085 | 0.8928 |
| Specificity | 0.9545 | 0.8747 | 0.9844 |
| Positive Predictive Value | 0.9474 | 0.8563 | 0.9819 |
| Negative Predictive Value | 0.84 | 0.7408 | 0.906 |
| Positive Likelihood Ratio | 18 | 5.9237 | 54.6951 |
| Negative Likelihood Ratio | 0.1905 | 0.1139 | 0.3186 |

The sensitivity of the **NativeThiol** test is **81.8%**. The probability of the test being positive is **81.8%**, when the disease is present.

The specificity of the **NativeThiol** test is **95.5%**. The probability of the test being negative is **95.5%**, when the disease is not present.

The positive predictive value of the **NativeThiol** test is **94.7%**. The probability of the presence of the disease is **94.7%**, when the test is positive.

The negative predictive value of the **NativeThiol** test is **84.0%**. The probability of the absence of the disease is **84.0%**, when the test is negative.

The positive likelihood ratio of the **NativeThiol** test is **18.000**. The ratio between the probability of a positive test result given the presence of the disease and the probability of a positive test result given the absence of the disease is **18.000**.

The negative likelihood ratio of the **NativeThiol** test is **0.191**. The ratio between the probability of a negative test result given the presence of the disease and the probability of a negative test result given the absence of the disease is **0.191**.

**Show**

**Marker:** Disulfide

**Descriptive Statistics**

| **Group** | **N** | **Mean** | **Std.Dev.** | **S.E.Mean** | **Mean (Lower)** | **Mean (Upper)** |
| --- | --- | --- | --- | --- | --- | --- |
| Control | 66 | 30.25 | 13.7514 | 1.6927 | 26.8695 | 33.6305 |
| vertigo | 66 | 104.2227 | 41.224 | 5.0743 | 94.0886 | 114.3568 |

**Test Results & Statistics**

**ROC statistics**

| **AUC** | **S.E.** | **Lower limit** | **Upper limit** | **z value** | **p value** |
| --- | --- | --- | --- | --- | --- |
| 0.996 | 0.003 | 0.9647 | 1 | 165.3105 | <0.001 |

When two observations are randomly selected, the test result of an observation with the disease is **99.6%** more likely to be positive than the test result of an observation without the disease.

**ROC coordinates**

| **Cut point** | **FPR** | **TPR** | **FNR** | **TNR** |
| --- | --- | --- | --- | --- |
| >=12.3 | 1 | 1 | 0 | 0 |
| >=13.4 | 0.9394 | 1 | 0 | 0.0606 |
| >=18.6 | 0.8939 | 1 | 0 | 0.1061 |
| >=19.9 | 0.8182 | 1 | 0 | 0.1818 |
| >=20 | 0.7879 | 1 | 0 | 0.2121 |
| >=20.4 | 0.7727 | 1 | 0 | 0.2273 |
| >=21.2 | 0.7424 | 1 | 0 | 0.2576 |
| >=22 | 0.7121 | 1 | 0 | 0.2879 |
| >=22.2 | 0.6818 | 1 | 0 | 0.3182 |
| >=22.6 | 0.6212 | 1 | 0 | 0.3788 |
| >=24.1 | 0.5606 | 1 | 0 | 0.4394 |
| >=28.3 | 0.4848 | 1 | 0 | 0.5152 |
| >=29.5 | 0.4697 | 1 | 0 | 0.5303 |
| >=30.8 | 0.4091 | 1 | 0 | 0.5909 |
| >=31.7 | 0.3636 | 1 | 0 | 0.6364 |
| >=32.7 | 0.3485 | 1 | 0 | 0.6515 |
| >=34.7 | 0.303 | 1 | 0 | 0.697 |
| >=39.4 | 0.2576 | 1 | 0 | 0.7424 |
| >=40.2 | 0.2424 | 1 | 0 | 0.7576 |
| >=40.6 | 0.2273 | 1 | 0 | 0.7727 |
| >=41.2 | 0.2121 | 1 | 0 | 0.7879 |
| >=41.9 | 0.197 | 1 | 0 | 0.803 |
| >=47 | 0.1667 | 1 | 0 | 0.8333 |
| >=47.3 | 0.1515 | 1 | 0 | 0.8485 |
| >=48.9 | 0.1515 | 0.9848 | 0.0152 | 0.8485 |
| >=49.8 | 0.1364 | 0.9848 | 0.0152 | 0.8636 |
| >=52.7 | 0.1212 | 0.9848 | 0.0152 | 0.8788 |
| >=56.6 | 0.1061 | 0.9848 | 0.0152 | 0.8939 |
| >=57.5 | 0.0909 | 0.9697 | 0.0303 | 0.9091 |
| >=58.6 | 0.0758 | 0.9697 | 0.0303 | 0.9242 |
| >=59.6 | 0.0455 | 0.9697 | 0.0303 | 0.9545 |
| >=60.4 | 0.0152 | 0.9697 | 0.0303 | 0.9848 |
| >=60.7 | 0.0152 | 0.9545 | 0.0455 | 0.9848 |
| >=61.2 | 0 | 0.9545 | 0.0455 | 1 |
| >=62 | 0 | 0.9394 | 0.0606 | 1 |
| >=62.9 | 0 | 0.9242 | 0.0758 | 1 |
| >=63.2 | 0 | 0.9091 | 0.0909 | 1 |
| >=63.6 | 0 | 0.8939 | 0.1061 | 1 |
| >=64.3 | 0 | 0.8636 | 0.1364 | 1 |
| >=65 | 0 | 0.8485 | 0.1515 | 1 |
| >=67.6 | 0 | 0.8333 | 0.1667 | 1 |
| >=67.8 | 0 | 0.8182 | 0.1818 | 1 |
| >=75.5 | 0 | 0.803 | 0.197 | 1 |
| >=75.7 | 0 | 0.7727 | 0.2273 | 1 |
| >=76 | 0 | 0.7576 | 0.2424 | 1 |
| >=77 | 0 | 0.7424 | 0.2576 | 1 |
| >=79.2 | 0 | 0.7273 | 0.2727 | 1 |
| >=82.6 | 0 | 0.7121 | 0.2879 | 1 |
| >=84.1 | 0 | 0.697 | 0.303 | 1 |
| >=84.7 | 0 | 0.6818 | 0.3182 | 1 |
| >=86.3 | 0 | 0.6667 | 0.3333 | 1 |
| >=87.8 | 0 | 0.6364 | 0.3636 | 1 |
| >=88.7 | 0 | 0.6212 | 0.3788 | 1 |
| >=90 | 0 | 0.6061 | 0.3939 | 1 |
| >=90.9 | 0 | 0.5909 | 0.4091 | 1 |
| >=91.1 | 0 | 0.5758 | 0.4242 | 1 |
| >=91.3 | 0 | 0.5606 | 0.4394 | 1 |
| >=92.1 | 0 | 0.5455 | 0.4545 | 1 |
| >=94.5 | 0 | 0.5303 | 0.4697 | 1 |
| >=94.7 | 0 | 0.5152 | 0.4848 | 1 |
| >=95.2 | 0 | 0.5 | 0.5 | 1 |
| >=95.3 | 0 | 0.4848 | 0.5152 | 1 |
| >=97.9 | 0 | 0.4697 | 0.5303 | 1 |
| >=99.6 | 0 | 0.4545 | 0.5455 | 1 |
| >=100.7 | 0 | 0.4394 | 0.5606 | 1 |
| >=103.7 | 0 | 0.4242 | 0.5758 | 1 |
| >=104.4 | 0 | 0.4091 | 0.5909 | 1 |
| >=106.9 | 0 | 0.3939 | 0.6061 | 1 |
| >=107.2 | 0 | 0.3788 | 0.6212 | 1 |
| >=107.6 | 0 | 0.3636 | 0.6364 | 1 |
| >=111 | 0 | 0.3485 | 0.6515 | 1 |
| >=111.1 | 0 | 0.3333 | 0.6667 | 1 |
| >=112.1 | 0 | 0.3182 | 0.6818 | 1 |
| >=114.1 | 0 | 0.303 | 0.697 | 1 |
| >=115.4 | 0 | 0.2879 | 0.7121 | 1 |
| >=117.5 | 0 | 0.2727 | 0.7273 | 1 |
| >=117.6 | 0 | 0.2576 | 0.7424 | 1 |
| >=124.3 | 0 | 0.2424 | 0.7576 | 1 |
| >=126.6 | 0 | 0.2273 | 0.7727 | 1 |
| >=126.7 | 0 | 0.2121 | 0.7879 | 1 |
| >=131.3 | 0 | 0.197 | 0.803 | 1 |
| >=132 | 0 | 0.1818 | 0.8182 | 1 |
| >=133.6 | 0 | 0.1667 | 0.8333 | 1 |
| >=139.7 | 0 | 0.1515 | 0.8485 | 1 |
| >=152.6 | 0 | 0.1364 | 0.8636 | 1 |
| >=153.2 | 0 | 0.1212 | 0.8788 | 1 |
| >=158 | 0 | 0.1061 | 0.8939 | 1 |
| >=159.1 | 0 | 0.0909 | 0.9091 | 1 |
| >=162.6 | 0 | 0.0758 | 0.9242 | 1 |
| >=173.2 | 0 | 0.0606 | 0.9394 | 1 |
| >=181.6 | 0 | 0.0455 | 0.9545 | 1 |
| >=192.8 | 0 | 0.0303 | 0.9697 | 1 |
| >=304.6 | 0 | 0.0152 | 0.9848 | 1 |
| Inf | 0 | 0 | 1 | 1 |

**Multiple comparisons**

**Test Direction**

**High:** Disulfide, OxidizedThiol

**Low:** Zn, Cu, TotalThiol, NativeThiol, ReducedThiol, ThiolOxRedRatio

| **Marker1 (A)** | **Marker2 (B)** | **AUC (I)** | **AUC (J)** | **Diff. (I,J)** | **S.E. (Diff(A,B))** | **z value** | **p value** | **p value (adj.)** |
| --- | --- | --- | --- | --- | --- | --- | --- | --- |
| Zn | Cu | 0.5077 | 0.6478 | -0.1402 | 0.0431 | -3.2532 | 0.001 | 0.032 |
| Zn | TotalThiol | 0.5077 | 0.7366 | -0.2289 | 0.0466 | -4.91 | <0.001 | <0.001 |
| Zn | NativeThiol | 0.5077 | 0.9403 | -0.4326 | 0.0473 | -9.1465 | <0.001 | <0.001 |
| Zn | Disulfide | 0.5077 | 0.996 | -0.4883 | 0.0498 | -9.8009 | <0.001 | <0.001 |
| Zn | ReducedThiol | 0.5077 | 0.9839 | -0.4762 | 0.0549 | -8.6708 | <0.001 | <0.001 |
| Zn | OxidizedThiol | 0.5077 | 0.9991 | -0.4914 | 0.0517 | -9.4986 | <0.001 | <0.001 |
| Zn | ThiolOxRedRatio | 0.5077 | 0.9986 | -0.4909 | 0.0516 | -9.5156 | <0.001 | <0.001 |
| Cu | TotalThiol | 0.6478 | 0.7366 | -0.0887 | 0.0268 | -3.316 | <0.001 | 0.026 |
| Cu | NativeThiol | 0.6478 | 0.9403 | -0.2925 | 0.0386 | -7.584 | <0.001 | <0.001 |
| Cu | Disulfide | 0.6478 | 0.996 | -0.3481 | 0.0506 | -6.8836 | <0.001 | <0.001 |
| Cu | ReducedThiol | 0.6478 | 0.9839 | -0.3361 | 0.0512 | -6.5646 | <0.001 | <0.001 |
| Cu | OxidizedThiol | 0.6478 | 0.9991 | -0.3512 | 0.046 | -7.6314 | <0.001 | <0.001 |
| Cu | ThiolOxRedRatio | 0.6478 | 0.9986 | -0.3508 | 0.0486 | -7.2211 | <0.001 | <0.001 |
| TotalThiol | NativeThiol | 0.7366 | 0.9403 | -0.2037 | 0.0333 | -6.1134 | <0.001 | <0.001 |
| TotalThiol | Disulfide | 0.7366 | 0.996 | -0.2594 | 0.0447 | -5.7976 | <0.001 | <0.001 |
| TotalThiol | ReducedThiol | 0.7366 | 0.9839 | -0.2474 | 0.0495 | -4.9946 | <0.001 | <0.001 |
| TotalThiol | OxidizedThiol | 0.7366 | 0.9991 | -0.2625 | 0.0441 | -5.9496 | <0.001 | <0.001 |
| TotalThiol | ThiolOxRedRatio | 0.7366 | 0.9986 | -0.2621 | 0.0458 | -5.7165 | <0.001 | <0.001 |
| NativeThiol | Disulfide | 0.9403 | 0.996 | -0.0557 | 0.0207 | -2.6887 | 0.007 | 0.201 |
| NativeThiol | ReducedThiol | 0.9403 | 0.9839 | -0.0436 | 0.0242 | -1.8039 | 0.071 | 1.000 |
| NativeThiol | OxidizedThiol | 0.9403 | 0.9991 | -0.0588 | 0.0195 | -3.0065 | 0.003 | 0.074 |
| NativeThiol | ThiolOxRedRatio | 0.9403 | 0.9986 | -0.0583 | 0.02 | -2.9097 | 0.004 | 0.101 |
| Disulfide | ReducedThiol | 0.996 | 0.9839 | 0.0121 | 0.0149 | 0.8072 | 0.420 | 1.000 |
| Disulfide | OxidizedThiol | 0.996 | 0.9991 | -0.0031 | 0.0023 | -1.3675 | 0.171 | 1.000 |
| Disulfide | ThiolOxRedRatio | 0.996 | 0.9986 | -0.0026 | 0.0026 | -1.0336 | 0.301 | 1.000 |
| ReducedThiol | OxidizedThiol | 0.9839 | 0.9991 | -0.0152 | 0.0153 | -0.9934 | 0.321 | 1.000 |
| ReducedThiol | ThiolOxRedRatio | 0.9839 | 0.9986 | -0.0147 | 0.0147 | -0.9998 | 0.317 | 1.000 |
| OxidizedThiol | ThiolOxRedRatio | 0.9991 | 0.9986 | 0.0005 | 0.0007 | 0.677 | 0.498 | 1.000 |

There is a statistically significant difference between the area under the ROC curves of **Cu** and **Zn** markers. The AUC level of **Cu** marker **(0.6478)** is found to be higher than the AUC level of **Zn** marker **(0.5077)**.

**Cutoff point and diagnostic statistics**

Method: Youden

Criterion: 0.9545

Cutoff: >=60.4

Positive class: vertigo

| **Measures** | **Value** | **Lower limit** | **Upper limit** |
| --- | --- | --- | --- |
| Sensitivity | 0.9697 | 0.8961 | 0.9917 |
| Specificity | 0.9848 | 0.919 | 0.9973 |
| Positive Predictive Value | 0.9846 | 0.9179 | 0.9973 |
| Negative Predictive Value | 0.9701 | 0.8975 | 0.9918 |
| Positive Likelihood Ratio | 64 | 9.1464 | 447.8282 |
| Negative Likelihood Ratio | 0.0308 | 0.0079 | 0.1205 |

The sensitivity of the **Disulfide** test is **97.0%**. The probability of the test being positive is **97.0%**, when the disease is present.

The specificity of the **Disulfide** test is **98.5%**. The probability of the test being negative is **98.5%**, when the disease is not present.

The positive predictive value of the **Disulfide** test is **98.5%**. The probability of the presence of the disease is **98.5%**, when the test is positive.

The negative predictive value of the **Disulfide** test is **97.0%**. The probability of the absence of the disease is **97.0%**, when the test is negative.

The positive likelihood ratio of the **Disulfide** test is **64.000**. The ratio between the probability of a positive test result given the presence of the disease and the probability of a positive test result given the absence of the disease is **64.000**.

The negative likelihood ratio of the **Disulfide** test is **0.031**. The ratio between the probability of a negative test result given the presence of the disease and the probability of a negative test result given the absence of the disease is **0.031**.

Method: Youden

Criterion: 0.9545

Cutoff: >=61.2

Positive class: vertigo

| **Measures** | **Value** | **Lower limit** | **Upper limit** |
| --- | --- | --- | --- |
| Sensitivity | 0.9545 | 0.8747 | 0.9844 |
| Specificity | 1 | 0.945 | 1 |
| Positive Predictive Value | 1 | 0.9425 | 1 |
| Negative Predictive Value | 0.9565 | 0.8798 | 0.9851 |
| Positive Likelihood Ratio | Inf |  | Inf |
| Negative Likelihood Ratio | 0.0455 | 0.015 | 0.1373 |

The sensitivity of the **Disulfide** test is **95.5%**. The probability of the test being positive is **95.5%**, when the disease is present.

The specificity of the **Disulfide** test is **100.0%**. The probability of the test being negative is **100.0%**, when the disease is not present.

The positive predictive value of the **Disulfide** test is **100.0%**. The probability of the presence of the disease is **100.0%**, when the test is positive.

The negative predictive value of the **Disulfide** test is **95.7%**. The probability of the absence of the disease is **95.7%**, when the test is negative.

The positive likelihood ratio of the **Disulfide** test is **NaN**. The ratio between the probability of a positive test result given the presence of the disease and the probability of a positive test result given the absence of the disease is **NaN**.

The negative likelihood ratio of the **Disulfide** test is **0.045**. The ratio between the probability of a negative test result given the presence of the disease and the probability of a negative test result given the absence of the disease is **0.045**.

**Show**

**Marker:** ReducedThiol

**Descriptive Statistics**

| **Group** | **N** | **Mean** | **Std.Dev.** | **S.E.Mean** | **Mean (Lower)** | **Mean (Upper)** |
| --- | --- | --- | --- | --- | --- | --- |
| Control | 66 | 89.9076 | 4.9571 | 0.6102 | 88.689 | 91.1262 |
| vertigo | 66 | 61.747 | 9.5312 | 1.1732 | 59.4039 | 64.09 |

**Test Results & Statistics**

**ROC statistics**

| **AUC** | **S.E.** | **Lower limit** | **Upper limit** | **z value** | **p value** |
| --- | --- | --- | --- | --- | --- |
| 0.9839 | 0.0152 | 0.9449 | 0.9979 | 31.8966 | <0.001 |

When two observations are randomly selected, the test result of an observation with the disease is **98.4%** more likely to be positive than the test result of an observation without the disease.

**ROC coordinates**

| **Cut point** | **FPR** | **TPR** | **FNR** | **TNR** |
| --- | --- | --- | --- | --- |
| -Inf | 0 | 0 | 1 | 1 |
| <=34.5 | 0 | 0.0152 | 0.9848 | 1 |
| <=40 | 0 | 0.0303 | 0.9697 | 1 |
| <=42.6 | 0 | 0.0455 | 0.9545 | 1 |
| <=43.1 | 0 | 0.0606 | 0.9394 | 1 |
| <=49.1 | 0 | 0.0758 | 0.9242 | 1 |
| <=51.2 | 0 | 0.0909 | 0.9091 | 1 |
| <=51.7 | 0 | 0.1061 | 0.8939 | 1 |
| <=52.1 | 0 | 0.1212 | 0.8788 | 1 |
| <=52.5 | 0 | 0.1364 | 0.8636 | 1 |
| <=53 | 0 | 0.1667 | 0.8333 | 1 |
| <=55.3 | 0 | 0.1818 | 0.8182 | 1 |
| <=56 | 0 | 0.197 | 0.803 | 1 |
| <=57 | 0 | 0.2121 | 0.7879 | 1 |
| <=57.1 | 0 | 0.2273 | 0.7727 | 1 |
| <=57.4 | 0 | 0.2424 | 0.7576 | 1 |
| <=57.5 | 0 | 0.2576 | 0.7424 | 1 |
| <=57.7 | 0 | 0.2727 | 0.7273 | 1 |
| <=57.8 | 0 | 0.2879 | 0.7121 | 1 |
| <=57.9 | 0 | 0.303 | 0.697 | 1 |
| <=58 | 0 | 0.3182 | 0.6818 | 1 |
| <=58.1 | 0 | 0.3333 | 0.6667 | 1 |
| <=58.6 | 0 | 0.3485 | 0.6515 | 1 |
| <=58.8 | 0 | 0.3636 | 0.6364 | 1 |
| <=59.3 | 0 | 0.3788 | 0.6212 | 1 |
| <=59.6 | 0 | 0.3939 | 0.6061 | 1 |
| <=59.8 | 0 | 0.4242 | 0.5758 | 1 |
| <=60 | 0 | 0.4394 | 0.5606 | 1 |
| <=60.5 | 0 | 0.4545 | 0.5455 | 1 |
| <=60.6 | 0 | 0.4697 | 0.5303 | 1 |
| <=61.4 | 0 | 0.4848 | 0.5152 | 1 |
| <=61.5 | 0 | 0.5 | 0.5 | 1 |
| <=61.9 | 0 | 0.5152 | 0.4848 | 1 |
| <=62 | 0 | 0.5303 | 0.4697 | 1 |
| <=62.2 | 0 | 0.5455 | 0.4545 | 1 |
| <=62.6 | 0 | 0.5606 | 0.4394 | 1 |
| <=63 | 0 | 0.5758 | 0.4242 | 1 |
| <=63.3 | 0 | 0.5909 | 0.4091 | 1 |
| <=65 | 0 | 0.6061 | 0.3939 | 1 |
| <=65.3 | 0 | 0.6364 | 0.3636 | 1 |
| <=66.2 | 0 | 0.6667 | 0.3333 | 1 |
| <=66.4 | 0 | 0.6818 | 0.3182 | 1 |
| <=66.8 | 0 | 0.697 | 0.303 | 1 |
| <=67 | 0 | 0.7121 | 0.2879 | 1 |
| <=67.1 | 0 | 0.7273 | 0.2727 | 1 |
| <=67.4 | 0 | 0.7424 | 0.2576 | 1 |
| <=67.5 | 0 | 0.7576 | 0.2424 | 1 |
| <=67.9 | 0 | 0.7727 | 0.2273 | 1 |
| <=68 | 0 | 0.7879 | 0.2121 | 1 |
| <=68.1 | 0 | 0.803 | 0.197 | 1 |
| <=68.3 | 0 | 0.8182 | 0.1818 | 1 |
| <=68.5 | 0 | 0.8333 | 0.1667 | 1 |
| <=68.6 | 0 | 0.8485 | 0.1515 | 1 |
| <=68.7 | 0 | 0.8636 | 0.1364 | 1 |
| <=69.8 | 0 | 0.8788 | 0.1212 | 1 |
| <=70.2 | 0 | 0.9091 | 0.0909 | 1 |
| <=70.7 | 0 | 0.9242 | 0.0758 | 1 |
| <=72 | 0 | 0.9394 | 0.0606 | 1 |
| <=72.6 | 0 | 0.9545 | 0.0455 | 1 |
| <=73.5 | 0 | 0.9697 | 0.0303 | 1 |
| <=75.5 | 0.0303 | 0.9697 | 0.0303 | 0.9697 |
| <=80 | 0.0606 | 0.9697 | 0.0303 | 0.9394 |
| <=80.9 | 0.0606 | 0.9848 | 0.0152 | 0.9394 |
| <=82 | 0.0758 | 0.9848 | 0.0152 | 0.9242 |
| <=82.9 | 0.0909 | 0.9848 | 0.0152 | 0.9091 |
| <=83 | 0.1061 | 0.9848 | 0.0152 | 0.8939 |
| <=83.1 | 0.1212 | 0.9848 | 0.0152 | 0.8788 |
| <=83.5 | 0.1364 | 0.9848 | 0.0152 | 0.8636 |
| <=84.2 | 0.1515 | 0.9848 | 0.0152 | 0.8485 |
| <=84.6 | 0.1667 | 0.9848 | 0.0152 | 0.8333 |
| <=85.3 | 0.197 | 0.9848 | 0.0152 | 0.803 |
| <=85.7 | 0.2121 | 0.9848 | 0.0152 | 0.7879 |
| <=86.6 | 0.2273 | 0.9848 | 0.0152 | 0.7727 |
| <=87 | 0.2424 | 0.9848 | 0.0152 | 0.7576 |
| <=87.1 | 0.2576 | 0.9848 | 0.0152 | 0.7424 |
| <=87.3 | 0.303 | 0.9848 | 0.0152 | 0.697 |
| <=87.5 | 0.3182 | 0.9848 | 0.0152 | 0.6818 |
| <=87.8 | 0.3333 | 0.9848 | 0.0152 | 0.6667 |
| <=87.9 | 0.3788 | 0.9848 | 0.0152 | 0.6212 |
| <=90.3 | 0.4242 | 0.9848 | 0.0152 | 0.5758 |
| <=90.6 | 0.4394 | 0.9848 | 0.0152 | 0.5606 |
| <=91.1 | 0.4848 | 0.9848 | 0.0152 | 0.5152 |
| <=91.6 | 0.5152 | 0.9848 | 0.0152 | 0.4848 |
| <=92.5 | 0.6061 | 0.9848 | 0.0152 | 0.3939 |
| <=93 | 0.6364 | 0.9848 | 0.0152 | 0.3636 |
| <=93.2 | 0.6515 | 0.9848 | 0.0152 | 0.3485 |
| <=93.3 | 0.7273 | 0.9848 | 0.0152 | 0.2727 |
| <=93.7 | 0.7576 | 0.9848 | 0.0152 | 0.2424 |
| <=93.9 | 0.8182 | 0.9848 | 0.0152 | 0.1818 |
| <=94.2 | 0.8939 | 0.9848 | 0.0152 | 0.1061 |
| <=95.4 | 0.9394 | 0.9848 | 0.0152 | 0.0606 |
| <=95.7 | 1 | 0.9848 | 0.0152 | 0 |
| <=99.6 | 1 | 1 | 0 | 0 |

**Multiple comparisons**

**Test Direction**

**High:** Disulfide, OxidizedThiol

**Low:** Zn, Cu, TotalThiol, NativeThiol, ReducedThiol, ThiolOxRedRatio

| **Marker1 (A)** | **Marker2 (B)** | **AUC (I)** | **AUC (J)** | **Diff. (I,J)** | **S.E. (Diff(A,B))** | **z value** | **p value** | **p value (adj.)** |
| --- | --- | --- | --- | --- | --- | --- | --- | --- |
| Zn | Cu | 0.5077 | 0.6478 | -0.1402 | 0.0431 | -3.2532 | 0.001 | 0.032 |
| Zn | TotalThiol | 0.5077 | 0.7366 | -0.2289 | 0.0466 | -4.91 | <0.001 | <0.001 |
| Zn | NativeThiol | 0.5077 | 0.9403 | -0.4326 | 0.0473 | -9.1465 | <0.001 | <0.001 |
| Zn | Disulfide | 0.5077 | 0.996 | -0.4883 | 0.0498 | -9.8009 | <0.001 | <0.001 |
| Zn | ReducedThiol | 0.5077 | 0.9839 | -0.4762 | 0.0549 | -8.6708 | <0.001 | <0.001 |
| Zn | OxidizedThiol | 0.5077 | 0.9991 | -0.4914 | 0.0517 | -9.4986 | <0.001 | <0.001 |
| Zn | ThiolOxRedRatio | 0.5077 | 0.9986 | -0.4909 | 0.0516 | -9.5156 | <0.001 | <0.001 |
| Cu | TotalThiol | 0.6478 | 0.7366 | -0.0887 | 0.0268 | -3.316 | <0.001 | 0.026 |
| Cu | NativeThiol | 0.6478 | 0.9403 | -0.2925 | 0.0386 | -7.584 | <0.001 | <0.001 |
| Cu | Disulfide | 0.6478 | 0.996 | -0.3481 | 0.0506 | -6.8836 | <0.001 | <0.001 |
| Cu | ReducedThiol | 0.6478 | 0.9839 | -0.3361 | 0.0512 | -6.5646 | <0.001 | <0.001 |
| Cu | OxidizedThiol | 0.6478 | 0.9991 | -0.3512 | 0.046 | -7.6314 | <0.001 | <0.001 |
| Cu | ThiolOxRedRatio | 0.6478 | 0.9986 | -0.3508 | 0.0486 | -7.2211 | <0.001 | <0.001 |
| TotalThiol | NativeThiol | 0.7366 | 0.9403 | -0.2037 | 0.0333 | -6.1134 | <0.001 | <0.001 |
| TotalThiol | Disulfide | 0.7366 | 0.996 | -0.2594 | 0.0447 | -5.7976 | <0.001 | <0.001 |
| TotalThiol | ReducedThiol | 0.7366 | 0.9839 | -0.2474 | 0.0495 | -4.9946 | <0.001 | <0.001 |
| TotalThiol | OxidizedThiol | 0.7366 | 0.9991 | -0.2625 | 0.0441 | -5.9496 | <0.001 | <0.001 |
| TotalThiol | ThiolOxRedRatio | 0.7366 | 0.9986 | -0.2621 | 0.0458 | -5.7165 | <0.001 | <0.001 |
| NativeThiol | Disulfide | 0.9403 | 0.996 | -0.0557 | 0.0207 | -2.6887 | 0.007 | 0.201 |
| NativeThiol | ReducedThiol | 0.9403 | 0.9839 | -0.0436 | 0.0242 | -1.8039 | 0.071 | 1.000 |
| NativeThiol | OxidizedThiol | 0.9403 | 0.9991 | -0.0588 | 0.0195 | -3.0065 | 0.003 | 0.074 |
| NativeThiol | ThiolOxRedRatio | 0.9403 | 0.9986 | -0.0583 | 0.02 | -2.9097 | 0.004 | 0.101 |
| Disulfide | ReducedThiol | 0.996 | 0.9839 | 0.0121 | 0.0149 | 0.8072 | 0.420 | 1.000 |
| Disulfide | OxidizedThiol | 0.996 | 0.9991 | -0.0031 | 0.0023 | -1.3675 | 0.171 | 1.000 |
| Disulfide | ThiolOxRedRatio | 0.996 | 0.9986 | -0.0026 | 0.0026 | -1.0336 | 0.301 | 1.000 |
| ReducedThiol | OxidizedThiol | 0.9839 | 0.9991 | -0.0152 | 0.0153 | -0.9934 | 0.321 | 1.000 |
| ReducedThiol | ThiolOxRedRatio | 0.9839 | 0.9986 | -0.0147 | 0.0147 | -0.9998 | 0.317 | 1.000 |
| OxidizedThiol | ThiolOxRedRatio | 0.9991 | 0.9986 | 0.0005 | 0.0007 | 0.677 | 0.498 | 1.000 |

There is a statistically significant difference between the area under the ROC curves of **Cu** and **Zn** markers. The AUC level of **Cu** marker **(0.6478)** is found to be higher than the AUC level of **Zn** marker **(0.5077)**.

**Cutoff point and diagnostic statistics**

Method: Youden

Criterion: 0.9697

Cutoff: <=73.5

Positive class: vertigo

| **Measures** | **Value** | **Lower limit** | **Upper limit** |
| --- | --- | --- | --- |
| Sensitivity | 0.9697 | 0.8961 | 0.9917 |
| Specificity | 1 | 0.945 | 1 |
| Positive Predictive Value | 1 | 0.9434 | 1 |
| Negative Predictive Value | 0.9706 | 0.899 | 0.9919 |
| Positive Likelihood Ratio | Inf |  | Inf |
| Negative Likelihood Ratio | 0.0303 | 0.0077 | 0.1186 |

The sensitivity of the **ReducedThiol** test is **97.0%**. The probability of the test being positive is **97.0%**, when the disease is present.

The specificity of the **ReducedThiol** test is **100.0%**. The probability of the test being negative is **100.0%**, when the disease is not present.

The positive predictive value of the **ReducedThiol** test is **100.0%**. The probability of the presence of the disease is **100.0%**, when the test is positive.

The negative predictive value of the **ReducedThiol** test is **97.1%**. The probability of the absence of the disease is **97.1%**, when the test is negative.

The positive likelihood ratio of the **ReducedThiol** test is **NaN**. The ratio between the probability of a positive test result given the presence of the disease and the probability of a positive test result given the absence of the disease is **NaN**.

The negative likelihood ratio of the **ReducedThiol** test is **0.030**. The ratio between the probability of a negative test result given the presence of the disease and the probability of a negative test result given the absence of the disease is **0.030**.

**Show**

**Marker:** OxidizedThiol

**Descriptive Statistics**

| **Group** | **N** | **Mean** | **Std.Dev.** | **S.E.Mean** | **Mean (Lower)** | **Mean (Upper)** |
| --- | --- | --- | --- | --- | --- | --- |
| Control | 66 | 5.0561 | 2.4823 | 0.3055 | 4.4458 | 5.6663 |
| vertigo | 66 | 19.3333 | 4.1894 | 0.5157 | 18.3034 | 20.3632 |

**Test Results & Statistics**

**ROC statistics**

| **AUC** | **S.E.** | **Lower limit** | **Upper limit** | **z value** | **p value** |
| --- | --- | --- | --- | --- | --- |
| 0.9991 | 0.001 | 0.9706 | 1 | 488.3805 | <0.001 |

When two observations are randomly selected, the test result of an observation with the disease is **99.9%** more likely to be positive than the test result of an observation without the disease.

**ROC coordinates**

| **Cut point** | **FPR** | **TPR** | **FNR** | **TNR** |
| --- | --- | --- | --- | --- |
| >=2.1 | 1 | 1 | 0 | 0 |
| >=2.3 | 0.9394 | 1 | 0 | 0.0606 |
| >=2.9 | 0.8939 | 1 | 0 | 0.1061 |
| >=3.1 | 0.8182 | 1 | 0 | 0.1818 |
| >=3.2 | 0.7576 | 1 | 0 | 0.2424 |
| >=3.4 | 0.7273 | 1 | 0 | 0.2727 |
| >=3.5 | 0.6364 | 1 | 0 | 0.3636 |
| >=3.7 | 0.6061 | 1 | 0 | 0.3939 |
| >=3.8 | 0.5455 | 1 | 0 | 0.4545 |
| >=4.2 | 0.5152 | 1 | 0 | 0.4848 |
| >=4.5 | 0.4848 | 1 | 0 | 0.5152 |
| >=4.7 | 0.4394 | 1 | 0 | 0.5606 |
| >=4.8 | 0.4242 | 1 | 0 | 0.5758 |
| >=6.1 | 0.3788 | 1 | 0 | 0.6212 |
| >=6.3 | 0.3182 | 1 | 0 | 0.6818 |
| >=6.4 | 0.303 | 1 | 0 | 0.697 |
| >=6.5 | 0.2576 | 1 | 0 | 0.7424 |
| >=6.7 | 0.2273 | 1 | 0 | 0.7727 |
| >=7.2 | 0.2121 | 1 | 0 | 0.7879 |
| >=7.3 | 0.197 | 1 | 0 | 0.803 |
| >=7.7 | 0.1667 | 1 | 0 | 0.8333 |
| >=7.9 | 0.1515 | 1 | 0 | 0.8485 |
| >=8.2 | 0.1364 | 1 | 0 | 0.8636 |
| >=8.5 | 0.1212 | 1 | 0 | 0.8788 |
| >=9 | 0.0758 | 1 | 0 | 0.9242 |
| >=9.5 | 0.0606 | 1 | 0 | 0.9394 |
| >=10 | 0.0606 | 0.9848 | 0.0152 | 0.9394 |
| >=12.3 | 0.0303 | 0.9848 | 0.0152 | 0.9697 |
| >=13.3 | 0 | 0.9848 | 0.0152 | 1 |
| >=13.7 | 0 | 0.9697 | 0.0303 | 1 |
| >=14 | 0 | 0.9545 | 0.0455 | 1 |
| >=14.1 | 0 | 0.9394 | 0.0606 | 1 |
| >=14.7 | 0 | 0.9242 | 0.0758 | 1 |
| >=14.9 | 0 | 0.9091 | 0.0909 | 1 |
| >=15.1 | 0 | 0.8788 | 0.1212 | 1 |
| >=15.6 | 0 | 0.8636 | 0.1364 | 1 |
| >=15.7 | 0 | 0.8485 | 0.1515 | 1 |
| >=15.9 | 0 | 0.8182 | 0.1818 | 1 |
| >=16 | 0 | 0.7879 | 0.2121 | 1 |
| >=16.2 | 0 | 0.7576 | 0.2424 | 1 |
| >=16.3 | 0 | 0.7424 | 0.2576 | 1 |
| >=16.5 | 0 | 0.7273 | 0.2727 | 1 |
| >=16.6 | 0 | 0.697 | 0.303 | 1 |
| >=16.8 | 0 | 0.6818 | 0.3182 | 1 |
| >=16.9 | 0 | 0.6667 | 0.3333 | 1 |
| >=17.3 | 0 | 0.6364 | 0.3636 | 1 |
| >=17.4 | 0 | 0.6212 | 0.3788 | 1 |
| >=17.5 | 0 | 0.6061 | 0.3939 | 1 |
| >=18.3 | 0 | 0.5909 | 0.4091 | 1 |
| >=18.5 | 0 | 0.5758 | 0.4242 | 1 |
| >=18.7 | 0 | 0.5606 | 0.4394 | 1 |
| >=18.9 | 0 | 0.5455 | 0.4545 | 1 |
| >=19 | 0 | 0.5303 | 0.4697 | 1 |
| >=19.2 | 0 | 0.5 | 0.5 | 1 |
| >=19.3 | 0 | 0.4848 | 0.5152 | 1 |
| >=19.7 | 0 | 0.4697 | 0.5303 | 1 |
| >=20 | 0 | 0.4394 | 0.5606 | 1 |
| >=20.1 | 0 | 0.4242 | 0.5758 | 1 |
| >=20.2 | 0 | 0.3939 | 0.6061 | 1 |
| >=20.4 | 0 | 0.3788 | 0.6212 | 1 |
| >=20.6 | 0 | 0.3636 | 0.6364 | 1 |
| >=20.7 | 0 | 0.3485 | 0.6515 | 1 |
| >=21 | 0 | 0.3333 | 0.6667 | 1 |
| >=21.1 | 0 | 0.303 | 0.697 | 1 |
| >=21.2 | 0 | 0.2727 | 0.7273 | 1 |
| >=21.3 | 0 | 0.2424 | 0.7576 | 1 |
| >=21.4 | 0 | 0.2273 | 0.7727 | 1 |
| >=21.5 | 0 | 0.2121 | 0.7879 | 1 |
| >=22 | 0 | 0.197 | 0.803 | 1 |
| >=22.3 | 0 | 0.1818 | 0.8182 | 1 |
| >=23.5 | 0 | 0.1667 | 0.8333 | 1 |
| >=23.7 | 0 | 0.1364 | 0.8636 | 1 |
| >=24 | 0 | 0.1212 | 0.8788 | 1 |
| >=24.2 | 0 | 0.1061 | 0.8939 | 1 |
| >=24.4 | 0 | 0.0909 | 0.9091 | 1 |
| >=25.4 | 0 | 0.0758 | 0.9242 | 1 |
| >=28.5 | 0 | 0.0606 | 0.9394 | 1 |
| >=28.7 | 0 | 0.0455 | 0.9545 | 1 |
| >=30 | 0 | 0.0303 | 0.9697 | 1 |
| >=32.7 | 0 | 0.0152 | 0.9848 | 1 |
| Inf | 0 | 0 | 1 | 1 |

**Multiple comparisons**

**Test Direction**

**High:** Disulfide, OxidizedThiol

**Low:** Zn, Cu, TotalThiol, NativeThiol, ReducedThiol, ThiolOxRedRatio

| **Marker1 (A)** | **Marker2 (B)** | **AUC (I)** | **AUC (J)** | **Diff. (I,J)** | **S.E. (Diff(A,B))** | **z value** | **p value** | **p value (adj.)** |
| --- | --- | --- | --- | --- | --- | --- | --- | --- |
| Zn | Cu | 0.5077 | 0.6478 | -0.1402 | 0.0431 | -3.2532 | 0.001 | 0.032 |
| Zn | TotalThiol | 0.5077 | 0.7366 | -0.2289 | 0.0466 | -4.91 | <0.001 | <0.001 |
| Zn | NativeThiol | 0.5077 | 0.9403 | -0.4326 | 0.0473 | -9.1465 | <0.001 | <0.001 |
| Zn | Disulfide | 0.5077 | 0.996 | -0.4883 | 0.0498 | -9.8009 | <0.001 | <0.001 |
| Zn | ReducedThiol | 0.5077 | 0.9839 | -0.4762 | 0.0549 | -8.6708 | <0.001 | <0.001 |
| Zn | OxidizedThiol | 0.5077 | 0.9991 | -0.4914 | 0.0517 | -9.4986 | <0.001 | <0.001 |
| Zn | ThiolOxRedRatio | 0.5077 | 0.9986 | -0.4909 | 0.0516 | -9.5156 | <0.001 | <0.001 |
| Cu | TotalThiol | 0.6478 | 0.7366 | -0.0887 | 0.0268 | -3.316 | <0.001 | 0.026 |
| Cu | NativeThiol | 0.6478 | 0.9403 | -0.2925 | 0.0386 | -7.584 | <0.001 | <0.001 |
| Cu | Disulfide | 0.6478 | 0.996 | -0.3481 | 0.0506 | -6.8836 | <0.001 | <0.001 |
| Cu | ReducedThiol | 0.6478 | 0.9839 | -0.3361 | 0.0512 | -6.5646 | <0.001 | <0.001 |
| Cu | OxidizedThiol | 0.6478 | 0.9991 | -0.3512 | 0.046 | -7.6314 | <0.001 | <0.001 |
| Cu | ThiolOxRedRatio | 0.6478 | 0.9986 | -0.3508 | 0.0486 | -7.2211 | <0.001 | <0.001 |
| TotalThiol | NativeThiol | 0.7366 | 0.9403 | -0.2037 | 0.0333 | -6.1134 | <0.001 | <0.001 |
| TotalThiol | Disulfide | 0.7366 | 0.996 | -0.2594 | 0.0447 | -5.7976 | <0.001 | <0.001 |
| TotalThiol | ReducedThiol | 0.7366 | 0.9839 | -0.2474 | 0.0495 | -4.9946 | <0.001 | <0.001 |
| TotalThiol | OxidizedThiol | 0.7366 | 0.9991 | -0.2625 | 0.0441 | -5.9496 | <0.001 | <0.001 |
| TotalThiol | ThiolOxRedRatio | 0.7366 | 0.9986 | -0.2621 | 0.0458 | -5.7165 | <0.001 | <0.001 |
| NativeThiol | Disulfide | 0.9403 | 0.996 | -0.0557 | 0.0207 | -2.6887 | 0.007 | 0.201 |
| NativeThiol | ReducedThiol | 0.9403 | 0.9839 | -0.0436 | 0.0242 | -1.8039 | 0.071 | 1.000 |
| NativeThiol | OxidizedThiol | 0.9403 | 0.9991 | -0.0588 | 0.0195 | -3.0065 | 0.003 | 0.074 |
| NativeThiol | ThiolOxRedRatio | 0.9403 | 0.9986 | -0.0583 | 0.02 | -2.9097 | 0.004 | 0.101 |
| Disulfide | ReducedThiol | 0.996 | 0.9839 | 0.0121 | 0.0149 | 0.8072 | 0.420 | 1.000 |
| Disulfide | OxidizedThiol | 0.996 | 0.9991 | -0.0031 | 0.0023 | -1.3675 | 0.171 | 1.000 |
| Disulfide | ThiolOxRedRatio | 0.996 | 0.9986 | -0.0026 | 0.0026 | -1.0336 | 0.301 | 1.000 |
| ReducedThiol | OxidizedThiol | 0.9839 | 0.9991 | -0.0152 | 0.0153 | -0.9934 | 0.321 | 1.000 |
| ReducedThiol | ThiolOxRedRatio | 0.9839 | 0.9986 | -0.0147 | 0.0147 | -0.9998 | 0.317 | 1.000 |
| OxidizedThiol | ThiolOxRedRatio | 0.9991 | 0.9986 | 0.0005 | 0.0007 | 0.677 | 0.498 | 1.000 |

There is a statistically significant difference between the area under the ROC curves of **Cu** and **Zn** markers. The AUC level of **Cu** marker **(0.6478)** is found to be higher than the AUC level of **Zn** marker **(0.5077)**.

**Cutoff point and diagnostic statistics**

Method: Youden

Criterion: 0.9848

Cutoff: >=13.3

Positive class: vertigo

| **Measures** | **Value** | **Lower limit** | **Upper limit** |
| --- | --- | --- | --- |
| Sensitivity | 0.9848 | 0.919 | 0.9973 |
| Specificity | 1 | 0.945 | 1 |
| Positive Predictive Value | 1 | 0.9442 | 1 |
| Negative Predictive Value | 0.9851 | 0.9202 | 0.9974 |
| Positive Likelihood Ratio | Inf |  | Inf |
| Negative Likelihood Ratio | 0.0152 | 0.0022 | 0.106 |

The sensitivity of the **OxidizedThiol** test is **98.5%**. The probability of the test being positive is **98.5%**, when the disease is present.

The specificity of the **OxidizedThiol** test is **100.0%**. The probability of the test being negative is **100.0%**, when the disease is not present.

The positive predictive value of the **OxidizedThiol** test is **100.0%**. The probability of the presence of the disease is **100.0%**, when the test is positive.

The negative predictive value of the **OxidizedThiol** test is **98.5%**. The probability of the absence of the disease is **98.5%**, when the test is negative.

The positive likelihood ratio of the **OxidizedThiol** test is **NaN**. The ratio between the probability of a positive test result given the presence of the disease and the probability of a positive test result given the absence of the disease is **NaN**.

The negative likelihood ratio of the **OxidizedThiol** test is **0.015**. The ratio between the probability of a negative test result given the presence of the disease and the probability of a negative test result given the absence of the disease is **0.015**.

**Show**

**Marker:** ThiolOxRedRatio

**Descriptive Statistics**

| **Group** | **N** | **Mean** | **Std.Dev.** | **S.E.Mean** | **Mean (Lower)** | **Mean (Upper)** |
| --- | --- | --- | --- | --- | --- | --- |
| Control | 66 | 2243.6712 | 1067.3318 | 131.3795 | 1981.2883 | 2506.0541 |
| vertigo | 66 | 343.8712 | 125.3679 | 15.4317 | 313.0519 | 374.6905 |

**Test Results & Statistics**

**ROC statistics**

| **AUC** | **S.E.** | **Lower limit** | **Upper limit** | **z value** | **p value** |
| --- | --- | --- | --- | --- | --- |
| 0.9986 | 0.0012 | 0.9697 | 1 | 400.8919 | <0.001 |

When two observations are randomly selected, the test result of an observation with the disease is **99.9%** more likely to be positive than the test result of an observation without the disease.

**ROC coordinates**

| **Cut point** | **FPR** | **TPR** | **FNR** | **TNR** |
| --- | --- | --- | --- | --- |
| -Inf | 0 | 0 | 1 | 1 |
| <=105.5 | 0 | 0.0152 | 0.9848 | 1 |
| <=133.2 | 0 | 0.0303 | 0.9697 | 1 |
| <=148.7 | 0 | 0.0455 | 0.9545 | 1 |
| <=151.5 | 0 | 0.0606 | 0.9394 | 1 |
| <=193.2 | 0 | 0.0758 | 0.9242 | 1 |
| <=209.8 | 0 | 0.0909 | 0.9091 | 1 |
| <=213.9 | 0 | 0.1061 | 0.8939 | 1 |
| <=217.2 | 0 | 0.1212 | 0.8788 | 1 |
| <=221.3 | 0 | 0.1364 | 0.8636 | 1 |
| <=225.8 | 0 | 0.1667 | 0.8333 | 1 |
| <=247.4 | 0 | 0.1818 | 0.8182 | 1 |
| <=254.2 | 0 | 0.197 | 0.803 | 1 |
| <=265 | 0 | 0.2121 | 0.7879 | 1 |
| <=266.6 | 0 | 0.2273 | 0.7727 | 1 |
| <=269.7 | 0 | 0.2424 | 0.7576 | 1 |
| <=271 | 0 | 0.2576 | 0.7424 | 1 |
| <=272.3 | 0 | 0.2727 | 0.7273 | 1 |
| <=273.8 | 0 | 0.2879 | 0.7121 | 1 |
| <=275 | 0 | 0.303 | 0.697 | 1 |
| <=275.8 | 0 | 0.3182 | 0.6818 | 1 |
| <=277 | 0 | 0.3333 | 0.6667 | 1 |
| <=282.8 | 0 | 0.3485 | 0.6515 | 1 |
| <=285.5 | 0 | 0.3636 | 0.6364 | 1 |
| <=290.9 | 0 | 0.3788 | 0.6212 | 1 |
| <=294.6 | 0 | 0.3939 | 0.6061 | 1 |
| <=297.3 | 0 | 0.4091 | 0.5909 | 1 |
| <=297.6 | 0 | 0.4242 | 0.5758 | 1 |
| <=299.7 | 0 | 0.4394 | 0.5606 | 1 |
| <=306.6 | 0 | 0.4545 | 0.5455 | 1 |
| <=308.1 | 0 | 0.4697 | 0.5303 | 1 |
| <=318.1 | 0 | 0.4848 | 0.5152 | 1 |
| <=319.7 | 0 | 0.5 | 0.5 | 1 |
| <=325.3 | 0 | 0.5152 | 0.4848 | 1 |
| <=325.8 | 0 | 0.5303 | 0.4697 | 1 |
| <=329 | 0 | 0.5455 | 0.4545 | 1 |
| <=334.2 | 0 | 0.5606 | 0.4394 | 1 |
| <=340.7 | 0 | 0.5758 | 0.4242 | 1 |
| <=345.2 | 0 | 0.5909 | 0.4091 | 1 |
| <=371.2 | 0 | 0.6061 | 0.3939 | 1 |
| <=376.2 | 0 | 0.6212 | 0.3788 | 1 |
| <=376.5 | 0 | 0.6364 | 0.3636 | 1 |
| <=391.1 | 0 | 0.6515 | 0.3485 | 1 |
| <=391.6 | 0 | 0.6667 | 0.3333 | 1 |
| <=395.1 | 0 | 0.6818 | 0.3182 | 1 |
| <=401.7 | 0 | 0.697 | 0.303 | 1 |
| <=407 | 0 | 0.7121 | 0.2879 | 1 |
| <=407.3 | 0 | 0.7273 | 0.2727 | 1 |
| <=413.1 | 0 | 0.7424 | 0.2576 | 1 |
| <=416.3 | 0 | 0.7576 | 0.2424 | 1 |
| <=423.1 | 0 | 0.7727 | 0.2273 | 1 |
| <=424.1 | 0 | 0.7879 | 0.2121 | 1 |
| <=427.2 | 0 | 0.803 | 0.197 | 1 |
| <=430.9 | 0 | 0.8182 | 0.1818 | 1 |
| <=435.9 | 0 | 0.8333 | 0.1667 | 1 |
| <=437.7 | 0 | 0.8485 | 0.1515 | 1 |
| <=439.1 | 0 | 0.8636 | 0.1364 | 1 |
| <=462.8 | 0 | 0.8788 | 0.1212 | 1 |
| <=470.4 | 0 | 0.8939 | 0.1061 | 1 |
| <=471 | 0 | 0.9091 | 0.0909 | 1 |
| <=482.1 | 0 | 0.9242 | 0.0758 | 1 |
| <=514.9 | 0 | 0.9394 | 0.0606 | 1 |
| <=529.2 | 0 | 0.9545 | 0.0455 | 1 |
| <=554.4 | 0 | 0.9697 | 0.0303 | 1 |
| <=616.1 | 0.0152 | 0.9697 | 0.0303 | 0.9848 |
| <=616.3 | 0.0303 | 0.9697 | 0.0303 | 0.9697 |
| <=703.7 | 0.0303 | 0.9848 | 0.0152 | 0.9697 |
| <=801.4 | 0.0455 | 0.9848 | 0.0152 | 0.9545 |
| <=801.5 | 0.0606 | 0.9848 | 0.0152 | 0.9394 |
| <=847.1 | 0.0606 | 1 | 0 | 0.9394 |
| <=908.3 | 0.0758 | 1 | 0 | 0.9242 |
| <=972.3 | 0.0909 | 1 | 0 | 0.9091 |
| <=977.7 | 0.1061 | 1 | 0 | 0.8939 |
| <=983 | 0.1212 | 1 | 0 | 0.8788 |
| <=1014.9 | 0.1364 | 1 | 0 | 0.8636 |
| <=1064.3 | 0.1515 | 1 | 0 | 0.8485 |
| <=1097.7 | 0.1667 | 1 | 0 | 0.8333 |
| <=1164.2 | 0.1818 | 1 | 0 | 0.8182 |
| <=1164.4 | 0.197 | 1 | 0 | 0.803 |
| <=1195.3 | 0.2121 | 1 | 0 | 0.7879 |
| <=1297 | 0.2273 | 1 | 0 | 0.7727 |
| <=1333.8 | 0.2424 | 1 | 0 | 0.7576 |
| <=1348.7 | 0.2576 | 1 | 0 | 0.7424 |
| <=1369.8 | 0.2727 | 1 | 0 | 0.7273 |
| <=1370.4 | 0.2879 | 1 | 0 | 0.7121 |
| <=1371.1 | 0.303 | 1 | 0 | 0.697 |
| <=1394.2 | 0.3182 | 1 | 0 | 0.6818 |
| <=1445.8 | 0.3333 | 1 | 0 | 0.6667 |
| <=1446.1 | 0.3485 | 1 | 0 | 0.6515 |
| <=1446.4 | 0.3636 | 1 | 0 | 0.6364 |
| <=1447.1 | 0.3788 | 1 | 0 | 0.6212 |
| <=1867.1 | 0.3939 | 1 | 0 | 0.6061 |
| <=1867.7 | 0.4091 | 1 | 0 | 0.5909 |
| <=1868.3 | 0.4242 | 1 | 0 | 0.5758 |
| <=1926.9 | 0.4394 | 1 | 0 | 0.5606 |
| <=2045.6 | 0.4545 | 1 | 0 | 0.5455 |
| <=2046.2 | 0.4697 | 1 | 0 | 0.5303 |
| <=2046.8 | 0.4848 | 1 | 0 | 0.5152 |
| <=2187.2 | 0.5 | 1 | 0 | 0.5 |
| <=2188.2 | 0.5152 | 1 | 0 | 0.4848 |
| <=2464.1 | 0.5303 | 1 | 0 | 0.4697 |
| <=2465 | 0.5455 | 1 | 0 | 0.4545 |
| <=2473.9 | 0.5606 | 1 | 0 | 0.4394 |
| <=2474.3 | 0.5758 | 1 | 0 | 0.4242 |
| <=2474.8 | 0.5909 | 1 | 0 | 0.4091 |
| <=2475.7 | 0.6061 | 1 | 0 | 0.3939 |
| <=2637.3 | 0.6212 | 1 | 0 | 0.3788 |
| <=2638.2 | 0.6364 | 1 | 0 | 0.3636 |
| <=2738.3 | 0.6515 | 1 | 0 | 0.3485 |
| <=2775.1 | 0.6667 | 1 | 0 | 0.3333 |
| <=2775.5 | 0.6818 | 1 | 0 | 0.3182 |
| <=2775.9 | 0.697 | 1 | 0 | 0.303 |
| <=2776.8 | 0.7121 | 1 | 0 | 0.2879 |
| <=2777.6 | 0.7273 | 1 | 0 | 0.2727 |
| <=2962.2 | 0.7424 | 1 | 0 | 0.2576 |
| <=2963.2 | 0.7576 | 1 | 0 | 0.2424 |
| <=3055 | 0.7727 | 1 | 0 | 0.2273 |
| <=3055.4 | 0.7879 | 1 | 0 | 0.2121 |
| <=3055.9 | 0.803 | 1 | 0 | 0.197 |
| <=3056.8 | 0.8182 | 1 | 0 | 0.1818 |
| <=3232.9 | 0.8333 | 1 | 0 | 0.1667 |
| <=3233.4 | 0.8485 | 1 | 0 | 0.1515 |
| <=3234 | 0.8636 | 1 | 0 | 0.1364 |
| <=3235 | 0.8788 | 1 | 0 | 0.1212 |
| <=3236.1 | 0.8939 | 1 | 0 | 0.1061 |
| <=4171.6 | 0.9091 | 1 | 0 | 0.0909 |
| <=4172.4 | 0.9242 | 1 | 0 | 0.0758 |
| <=4173.1 | 0.9394 | 1 | 0 | 0.0606 |
| <=4456.3 | 0.9545 | 1 | 0 | 0.0455 |
| <=4457.1 | 0.9697 | 1 | 0 | 0.0303 |
| <=4458 | 0.9848 | 1 | 0 | 0.0152 |
| <=4459.6 | 1 | 1 | 0 | 0 |

**Multiple comparisons**

**Test Direction**

**High:** Disulfide, OxidizedThiol

**Low:** Zn, Cu, TotalThiol, NativeThiol, ReducedThiol, ThiolOxRedRatio

| **Marker1 (A)** | **Marker2 (B)** | **AUC (I)** | **AUC (J)** | **Diff. (I,J)** | **S.E. (Diff(A,B))** | **z value** | **p value** | **p value (adj.)** |
| --- | --- | --- | --- | --- | --- | --- | --- | --- |
| Zn | Cu | 0.5077 | 0.6478 | -0.1402 | 0.0431 | -3.2532 | 0.001 | 0.032 |
| Zn | TotalThiol | 0.5077 | 0.7366 | -0.2289 | 0.0466 | -4.91 | <0.001 | <0.001 |
| Zn | NativeThiol | 0.5077 | 0.9403 | -0.4326 | 0.0473 | -9.1465 | <0.001 | <0.001 |
| Zn | Disulfide | 0.5077 | 0.996 | -0.4883 | 0.0498 | -9.8009 | <0.001 | <0.001 |
| Zn | ReducedThiol | 0.5077 | 0.9839 | -0.4762 | 0.0549 | -8.6708 | <0.001 | <0.001 |
| Zn | OxidizedThiol | 0.5077 | 0.9991 | -0.4914 | 0.0517 | -9.4986 | <0.001 | <0.001 |
| Zn | ThiolOxRedRatio | 0.5077 | 0.9986 | -0.4909 | 0.0516 | -9.5156 | <0.001 | <0.001 |
| Cu | TotalThiol | 0.6478 | 0.7366 | -0.0887 | 0.0268 | -3.316 | <0.001 | 0.026 |
| Cu | NativeThiol | 0.6478 | 0.9403 | -0.2925 | 0.0386 | -7.584 | <0.001 | <0.001 |
| Cu | Disulfide | 0.6478 | 0.996 | -0.3481 | 0.0506 | -6.8836 | <0.001 | <0.001 |
| Cu | ReducedThiol | 0.6478 | 0.9839 | -0.3361 | 0.0512 | -6.5646 | <0.001 | <0.001 |
| Cu | OxidizedThiol | 0.6478 | 0.9991 | -0.3512 | 0.046 | -7.6314 | <0.001 | <0.001 |
| Cu | ThiolOxRedRatio | 0.6478 | 0.9986 | -0.3508 | 0.0486 | -7.2211 | <0.001 | <0.001 |
| TotalThiol | NativeThiol | 0.7366 | 0.9403 | -0.2037 | 0.0333 | -6.1134 | <0.001 | <0.001 |
| TotalThiol | Disulfide | 0.7366 | 0.996 | -0.2594 | 0.0447 | -5.7976 | <0.001 | <0.001 |
| TotalThiol | ReducedThiol | 0.7366 | 0.9839 | -0.2474 | 0.0495 | -4.9946 | <0.001 | <0.001 |
| TotalThiol | OxidizedThiol | 0.7366 | 0.9991 | -0.2625 | 0.0441 | -5.9496 | <0.001 | <0.001 |
| TotalThiol | ThiolOxRedRatio | 0.7366 | 0.9986 | -0.2621 | 0.0458 | -5.7165 | <0.001 | <0.001 |
| NativeThiol | Disulfide | 0.9403 | 0.996 | -0.0557 | 0.0207 | -2.6887 | 0.007 | 0.201 |
| NativeThiol | ReducedThiol | 0.9403 | 0.9839 | -0.0436 | 0.0242 | -1.8039 | 0.071 | 1.000 |
| NativeThiol | OxidizedThiol | 0.9403 | 0.9991 | -0.0588 | 0.0195 | -3.0065 | 0.003 | 0.074 |
| NativeThiol | ThiolOxRedRatio | 0.9403 | 0.9986 | -0.0583 | 0.02 | -2.9097 | 0.004 | 0.101 |
| Disulfide | ReducedThiol | 0.996 | 0.9839 | 0.0121 | 0.0149 | 0.8072 | 0.420 | 1.000 |
| Disulfide | OxidizedThiol | 0.996 | 0.9991 | -0.0031 | 0.0023 | -1.3675 | 0.171 | 1.000 |
| Disulfide | ThiolOxRedRatio | 0.996 | 0.9986 | -0.0026 | 0.0026 | -1.0336 | 0.301 | 1.000 |
| ReducedThiol | OxidizedThiol | 0.9839 | 0.9991 | -0.0152 | 0.0153 | -0.9934 | 0.321 | 1.000 |
| ReducedThiol | ThiolOxRedRatio | 0.9839 | 0.9986 | -0.0147 | 0.0147 | -0.9998 | 0.317 | 1.000 |
| OxidizedThiol | ThiolOxRedRatio | 0.9991 | 0.9986 | 0.0005 | 0.0007 | 0.677 | 0.498 | 1.000 |

There is a statistically significant difference between the area under the ROC curves of **Cu** and **Zn** markers. The AUC level of **Cu** marker **(0.6478)** is found to be higher than the AUC level of **Zn** marker **(0.5077)**.

**Cutoff point and diagnostic statistics**

Method: Youden

Criterion: 0.9697

Cutoff: <=554.4

Positive class: vertigo

| **Measures** | **Value** | **Lower limit** | **Upper limit** |
| --- | --- | --- | --- |
| Sensitivity | 0.9697 | 0.8961 | 0.9917 |
| Specificity | 1 | 0.945 | 1 |
| Positive Predictive Value | 1 | 0.9434 | 1 |
| Negative Predictive Value | 0.9706 | 0.899 | 0.9919 |
| Positive Likelihood Ratio | Inf |  | Inf |
| Negative Likelihood Ratio | 0.0303 | 0.0077 | 0.1186 |

The sensitivity of the **ThiolOxRedRatio** test is **97.0%**. The probability of the test being positive is **97.0%**, when the disease is present.

The specificity of the **ThiolOxRedRatio** test is **100.0%**. The probability of the test being negative is **100.0%**, when the disease is not present.

The positive predictive value of the **ThiolOxRedRatio** test is **100.0%**. The probability of the presence of the disease is **100.0%**, when the test is positive.

The negative predictive value of the **ThiolOxRedRatio** test is **97.1%**. The probability of the absence of the disease is **97.1%**, when the test is negative.

The positive likelihood ratio of the **ThiolOxRedRatio** test is **NaN**. The ratio between the probability of a positive test result given the presence of the disease and the probability of a positive test result given the absence of the disease is **NaN**.

The negative likelihood ratio of the **ThiolOxRedRatio** test is **0.030**. The ratio between the probability of a negative test result given the presence of the disease and the probability of a negative test result given the absence of the disease is **0.030**.
