# Supplementary material for: An evaluation of trace elements and oxidative stress in patients with benign paroxysmal positional vertigo
Source: PLoS One. 2023 Feb 22;18(2):e0277960. doi: 10.1371/journal.pone.0277960 (PMC9946263; doi:10.1371/journal.pone.0277960)
Supplement: Attachment — Submitted filename: review result 1.docx [file pone.0277960.s004.docx]

An evaluation of trace elements and oxidative stress in patients with benign paroxysmal positional vertigo.

Authors resulted that low Cu and Zn levels decrease the antioxidant activity and increase the formation of BPPV and serum oxidative stress and trace elements play a role in the pathophysiology of BPPV. Outcomes of the present study would provide useful information for readers. However, data presentation is insufficient for acceptance without revision. The manuscript needs to be revised in the followings.

#Patients with a history of Meniere's disease, vestibular neuritis, acoustic neurinoma, sudden hearing loss, hearing loss on audiometric examination, history of head trauma, and having an otologic surgery were not included in the study.

It is said that MD and SD are caused by stress, too. Why did you exclude these diseases?

If you conclude that the onset of BPPV is due to a decrease in Cu and Zn, isn’t it necessary to compare BPPV and other vertiginous diseases?

Overdose of Zn over a long period of time reduces Cu absorption. Therefore, it may be a physiological reaction. How about this physiological reaction?

#Serum Zn and Cu values can give information about antioxidant capacity. Low serum Cu and Zn may contribute to the development of BPPV.

The relationship between changes in serum Zn and Cu levels and oxidative stress due to vitamin D deficiency is unclear. To compare the article, authors should provide actual data on this relationship.
