# Supplementary material for: An evaluation of trace elements and oxidative stress in patients with benign paroxysmal positional vertigo
Source: PLoS One. 2023 Feb 22;18(2):e0277960. doi: 10.1371/journal.pone.0277960 (PMC9946263; doi:10.1371/journal.pone.0277960)
Supplement: Attachment — Submitted filename: Response to Reviewers.docx [file pone.0277960.s005.docx]

Response to Reviewers

*Additional Staff Editor comments: We noted your manuscript is similar to this published work https://pubmed.ncbi.nlm.nih.gov/30256204/. Please note that our policy requires that related works must be discussed and justifications should be provided in terms of the differences and what contributions you study make to the community. Please discuss this related work in more details.

This article was mentioned in the discussion of our previous article. but it was mentioned in more detail on your suggestion.

‘’ However, there are very few studies on BPPV and TDH in the literature. Şahin E. et al. found significantly lower SH and high SD rates during an attack. Their study is similar to our study but our study is more comprehensive and valuable due to the large number of patients and the fact that we have measured zinc and copper (23). In addition, the results of Şahin et al.'s study are consistent with the results of our study and support our findings..’’

**#Patients with a history of Meniere's disease, vestibular neuritis, acoustic neurinoma, sudden hearing loss, hearing loss on audiometric examination, history of head trauma, and having an otologic surgery were not included in the study. It is said that MD and SD are caused by stress, too. Why did you exclude these diseases?

If you conclude that the onset of BPPV is due to a decrease in Cu and Zn, isn’t it necessary to compare BPPV and other vertiginous diseases?

It is known that endolymphatic hydrops rather than stress has a role in the etiology of Meniere's disease. this is far from the etiology of BPVV. In addition, since the etiologies of MD and SD were different from BPVV, these patients were evaluated within the differential diagnosis.

***If you conclude that the onset of BPPV is due to a decrease in Cu and Zn, isn’t it necessary to compare BPPV and other vertiginous diseases?

The onset of BPPV is associated with the decrease in Cu and Zn. Comparison of BPPV with other vertigo diseases can be made with additional and new studies. It may be suggested in the discussion to make new measurements to see if there is the same situation in diseases with similar etiology.

****Overdose of Zn over a long period of time reduces Cu absorption. Therefore, it may be a physiological reaction. How about this physiological reaction?

An overdose of Zn over a long period of time reduces Cu absorption. Therefore, copper may be low as a result of a physiological reaction. However, the low levels of zinc and copper in our study do not match the physiological picture. In addition, there was no use of high-dose zinc in our study.

*****#Serum Zn and Cu values can give information about antioxidant capacity. Low serum Cu and Zn may contribute to the development of BPPV. The relationship between changes in serum Zn and Cu levels and oxidative stress due to vitamin D deficiency is unclear. To compare the article, authors should provide actual data on this relationship.

In the study conducted by Socha et al., the decrease in zinc and Total antioxidant status in Alzheimer's patients compared to the control group also supports our study results (2) However, in our study, we could not measure serum calcium and vitamin D values in our patients due to financial reasons. And this topic was mentioned in the article.

1. Şahin E, Deveci İ, Dinç ME, Özker BY, Biçer C, Erel Ö. Oxidative Status in Patients with Benign Paroxysmal Positional Vertigo. J Int Adv Otol. 2018 Aug;14(2):299-303. doi: 10.5152/iao.2018.4756.

2. Socha K, Klimiuk K, Naliwajko SK, Soroczyńska J, Puścion-Jakubik A, Markiewicz-Żukowska R, Kochanowicz J. Dietary Habits, Selenium, Copper, Zinc and Total Antioxidant Status in Serum in Relation to Cognitive Functions of Patients with Alzheimer's Disease. Nutrients. 2021 Jan 20;13(2):287. doi: 10.3390/nu13020287.
